# Transcatheter Treatment of Bicuspid Aortic Valve Stenosis: From Observational Studies to Randomized Clinical Trials

**DOI:** 10.1016/j.shj.2025.100754

**Published:** 2025-11-10

**Authors:** Daryoush Samim, Matthias Siepe, Peter Jüni, Aakriti Gupta, Hasan Jilaihawi, Michael A. Borger, Raj R. Makkar, Martin B. Leon, Stephan Windecker

**Affiliations:** a Department of Cardiology, Bern University Hospital, Bern, Switzerland; b Clinical Trial Service Unit and Epidemiological Studies Unit, Nuffield Department of Population Health, University of Oxford, Oxford, UK; c Department of Cardiac Surgery, Bern University Hospital, Bern, Switzerland; d Smidt Heart Institute, Cedars-Sinai Medical Center, Los Angeles, California, USA; e University Clinic of Cardiac Surgery, Leipzig Heart Center, Leipzig, Germany; f Division of Cardiology, Columbia University Irving Medical Center/NewYork Presbyterian Hospital, New York, New York, USA; g Cardiovascular Research Foundation, New York, New York, USA

**Keywords:** Aortic stenosis, Aortic valve, Bicuspid, Randomized controlled trial, SAVR, Surgery, TAVI, TAVR, Transcatheter

## Abstract

Bicuspid aortic valve (BAV) disease is the most common congenital heart abnormality, which affects up to 2% of the population and significantly increases the lifetime risk of aortic stenosis and the need for valve replacement. While surgical aortic valve replacement (SAVR) remains the standard of care in low surgical-risk patients, transcatheter aortic valve implantation (TAVI) is increasingly used as a treatment alternative with favorable outcomes in well-selected BAV patients. To date, randomized controlled trials comparing TAVI and SAVR have excluded patients with BAV, except the UK TAVI and the more recent The NOrdic Aortic Valve IntervenTION-2 trial. Of note, in The NOrdic Aortic Valve IntervenTION-2 trial, there was a numerical imbalance in the BAV subgroup, with more events observed in the TAVI group than in the SAVR group for the composite outcome (all-cause mortality, stroke, and rehospitalization) at 1- and 3-year follow-up, although the risk estimates were imprecise due to the limited number of events. The upcoming TraNscatheter Aortic Valve Implantation versus surGical AorTic valvE replacement in patients with Bicuspid aortic valve stenosis (NAVIGATE Bicuspid) and Bicuspid aortic valve replacement: EvaLuatIon of transcathetEr VERsus Surgery (BELIEVERS) trials will directly compare TAVI and SAVR in BAV populations. These trials are designed to provide robust, adequately powered estimates of the comparative safety and effectiveness of both interventions over extended follow-up and are expected to inform future guideline recommendations in the setting of BAV anatomy.

This review summarizes current data on the use of TAVI in patients with severe BAV stenosis, evaluates anatomical and procedural complexities, and outlines the design and rationale of upcoming randomized controlled trials addressing this critical knowledge gap.

## Highlights

• Bicuspid aortic valve (BAV) is the most common congenital heart defect, affecting up to 2% of the population and increasing the lifetime risk of aortic stenosis and the need for aortic valve replacement.
• Surgical aortic valve replacement (SAVR) remains the standard of care in low surgical-risk BAV patients, but transcatheter aortic valve implantation (TAVI) is increasingly used in selected BAV patients.
• Anatomical and procedural challenges—such as raphe calcification, elliptical annuli, and aortopathy—require individualized CT-based planning and careful patient and device selection.
• The NOTION-2 trial provided the first randomized data in a subgroup of BAV patients, showing numerically higher but imprecise event rates after TAVI compared with SAVR.
• This review summarizes current evidence on TAVI in BAV stenosis and outlines the upcoming NAVIGATE Bicuspid and BELIEVERS trials, which will directly compare TAVI and SAVR in BAV populations using long-term follow-up and harmonized endpoint definitions to address current evidence gaps. These trials aim to deliver robust data on safety, effectiveness, and quality of life, shaping future clinical guidelines and treatment strategies for patients with BAV stenosis.

## Graphical abstract

Transcatheter treatment of bicuspid aortic valve (BAV) stenosis: From observational studies to randomized clinical trials. Abbreviations: CT, computed tomography; IPW, inverse probapbility weighting; MACE, major adverse cardiovascular events; PS, propensity score; RCT, randomized controlled trial; SAVR, surgical aortic valve replacement; TAV, tricuspid aortic valve; TAVI, transcatheter aortic valve implantation; TF, transfemoral.

**Figure:**
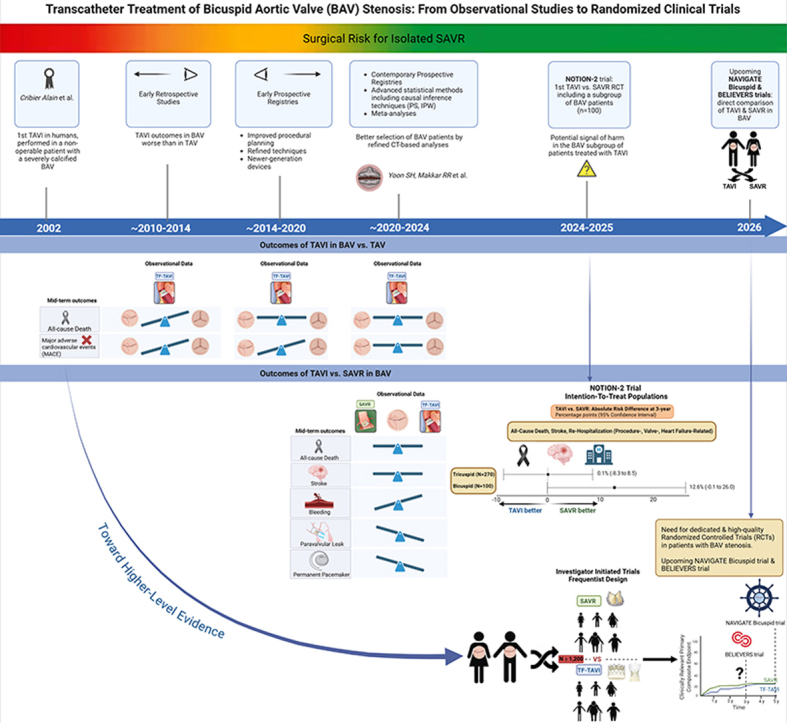

## Background

Aortic stenosis (AS), primarily caused by calcific degeneration of a native tricuspid (trileaflet) aortic valve (TAV) or bicuspid aortic valve (BAV),^1^^,^^2^ accounts for the largest number of deaths due to valvular heart disease in high-income countries^2^ and is the most common primary valve lesion requiring intervention in Europe and North America.^1^ Twenty-three years after the first transcatheter aortic valve implantation (TAVI) in humans, coincidentally performed in a patient with a severely calcified BAV,^3^ TAVI has emerged as a transformative alternative to surgical aortic valve replacement (SAVR) for the treatment of patients with severe AS.^4^

BAV, with a prevalence between 0.5 and 2.0% in the general population, is one of the most common congenital heart defects.^5^ Abnormal shear stress due to the underlying bicuspid morphology contributes to early valve degeneration and calcification, although some BAV patients present early in life with aortic insufficiency without calcification. Recent transcriptomic analyses have identified unique gene expression signatures associated with disease severity in BAV and TAV, with BAV stenosis being characterized by earlier onset and greater extent of calcification with upregulation of genes related to extracellular matrix remodeling and ossification, whereas TAV stenosis has been associated with evidence of stronger inflammatory signatures.^6^ Compared with trileaflet valves, BAV significantly increases the lifetime risk of developing AS.^5^^,^^7^ Patients with BAV face an estimated 70% lifetime risk of undergoing aortic valve surgery,^8^ often at a younger age compared with patients with trileaflet anatomy.^9^, ^10^, ^11^ BAV is present in up to 40% of patients undergoing SAVR,^12^ which remains the standard of care in low surgical-risk patients suffering from BAV stenosis according to the current 2025 European Society of Cardiology/European Association for Cardio-Thoracic Surgery (ESC/EACTS) and 2020 American College of Cardiology/American Heart Association (ACC/AHA) guidelines.^13^^,^^14^ However, the advent of TAVI along with rapid technological advancements during the past 2 decades^4^^,^^15^ has ignited a debate about optimal treatment strategy among patients with severe BAV stenosis.^16^ Over the last decade, the annual rate of patients with BAV undergoing TAVI has steadily increased in Europe and the United States, with recent studies reporting that BAV is present in ∼17% of patients undergoing TAVI in these regions.^17^^,^^18^

Eleven randomized controlled trials (RCTs), including 11,515 patients, have directly compared TAVI and SAVR across the spectrum of risk in patients with TAV AS to date. All RCTs comparing TAVI vs. SAVR have excluded patients with BAV, except the UK TAVI trial and more recently The NOrdic Aortic Valve IntervenTION (NOTION)-2 trial, over concerns related to anatomical and technical differences within this specific patient population. A synthesis of all RCTs in patients with TAV stenosis provides robust evidence that TAVI is superior to medical therapy alone in high-risk inoperable patients and has similar outcomes in terms of safety and effectiveness compared to SAVR in high-, intermediate-, and low surgical-risk patients at a follow-up duration of 5 to 10 years.^19^, ^20^, ^21^, ^22^, ^23^, ^24^, ^25^, ^26^, ^27^, ^28^, ^29^, ^30^, ^31^, ^32^

Owing to the increased prevalence of BAV stenosis in younger individuals, the expanding use of TAVI in lower-risk populations,^17^^,^^33^ and the inconclusive findings in the bicuspid subgroup of patients treated by TAVI in the recent NOTION-2 trial,^34^ a direct comparison between TAVI and SAVR in this population is required.

## Anatomical Considerations in Bicuspid Aortic Valve Disease

### Leaflet Morphology

BAV represents a wide range of abnormal aortic valve morphologies in which the valve consists of 2 functional cusps. The congenital fibrous ridge between the fused cusps is referred to as *raphe* and is frequently the location of progressive calcium deposition.^35^ Several classifications have been proposed, each with distinct strengths, limitations, and clinical applications. The “historical-surgical” classification by *Sievers* and *Schmidtke* (2007) categorizes BAV subtypes based on the number of raphes (0, 1, or 2).^35^ The “interventional” classification by *Jilaihawi* et al. (2016)^36^ is an imaging-based system derived from preprocedural TAVI computed tomography (CT) that classifies BAVs according to the number of functional commissures (bicommissural vs. tricommissural) and the presence or absence of a raphe. This classification challenges the traditional term “bicuspid,” proposing “bileaflet” as an alternative, since acquired commissural fusion may also result in 2 functional cusps. In addition, it emphasizes the interaction between TAVI prostheses and the aortic root, highlighting the procedural relevance of calcified raphes and commissural fusion, particularly in tricommissural anatomies, which represent acquired rather than congenital forms of BAV. The international consensus classification by *Michelena* et al. (2021)^37^ incorporates the number of sinuses, and the presence and extent of cusp fusion and describes a spectrum of the heterogeneous anatomy of BAV, from “almost tricuspid” to “perfect bicuspidity.” In this scheme, fused BAV is the most frequent phenotype (∼90-95% of all BAV)—most commonly left-right fusion (∼70-80%), followed by right-noncoronary cusp fusion (∼20-30%) and left-noncoronary cusp fusion (∼3-6%), with similar distributions in both sexes.^37^^,^^38^ Approximately 70% of fused BAVs exhibit a raphe, at least in Western populations, with a relatively more balanced frequency of raphe-type and non-raphe-type BAV seen in China.^39^ The 2-sinus BAV (∼5-7% of all BAV) consists of only 2 sinuses of Valsalva and 2 roughly equal-sized symmetrical cusps, with no raphe. The partial-fusion BAV of unknown prevalence demonstrates <50% fusion of 2 cusps (mini-raphe). Of note, *Sievers* type 2 (2 raphes) is rare (<1%) and reclassified as unicuspid in the international consensus BAV classification.^38^

### Aortopathy

BAV is associated with aortopathy—particularly ascending aortic dilatation—in up to 50% of patients, which increases the risk of aortic dissection.^40^, ^41^, ^42^, ^43^ BAV patients presenting with aortic insufficiency or “root phenotype” have a particularly increased risk of aortic dilation and acute aortic syndromes.^44^ Although some reports have suggested that growth of the ascending aorta and further dilatation may slow after TAVI treatment of BAV stenosis,^45^ larger ascending aortic diameters (≥40–45 mm) have been correlated in some studies with higher mortality and adverse outcomes,^46^^,^^47^ underscoring the need for thorough preprocedural CT assessment of the aorta, as well as careful TAVI procedural technique in the presence of aortopathy. Compared to TAV, BAV patients typically have larger annuli and sinuses of Valsalva, although this does not necessarily require more frequent prosthesis oversizing.^48^ BAV is also associated with coarctation of the aorta^43^ and a higher prevalence of a horizontal aorta (≥50° angulation), particularly in stenotic cases.^39^^,^^49^ Delivering TAVI devices through stented or surgically repaired coarctation is technically possible, and current data are reassuring.^50^

## Imaging and Procedural Planning in Bicuspid Aortic Valve Disease

**Figure 1:**
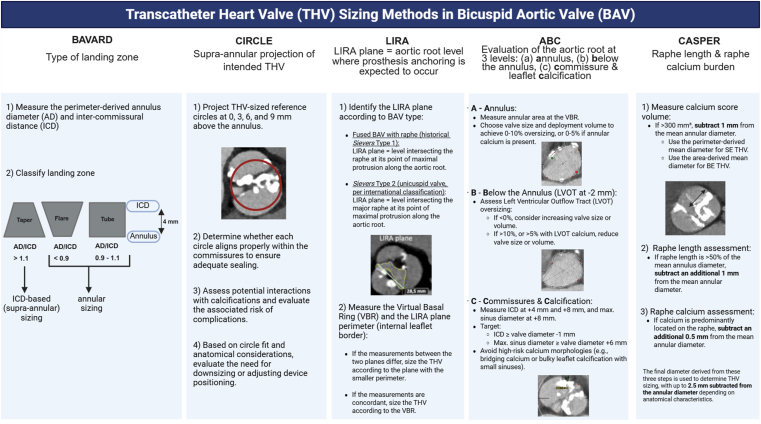
**Transcatheter heart valve (THV) sizing in bicuspid aortic valve (BAV).** Abbreviations: AD, annulus diameter; ICD, intercommissural distance; BE, balloon-expandable; LVOT, left ventricular outflow tract; SE, self-expanding; VBR, virtual basal ring.

Accurate CT-based transcatheter heart valve (THV) sizing is crucial to ensure procedural success in BAV due to the complex anatomy, characterized by raphe in most cases, asymmetric calcification pattern with variable extension into the left ventricular outflow tract, frequently elliptical annuli, and concomitant aortopathy. The BAV-associated aortic root may display tube-, flare-, or taper-shaped morphologies, with the narrowest point at either the annular (virtual basal ring) or supra-annular level. Alternative sizing strategies incorporate infra-annular (ABC method^51^) or supra-annular measurements (BAVARD^52^, Circle,^53^ LIRA^54^ and ABC^51^ methods, or multiplanar strategy^55^), and raphe and calcium burden assessment (CASPER^56^ method) (Figure 1). However, annular sizing remains the standard and most widely used approach. Based on reported difficulties with the reproducibility of annular measurements^57^ and in the absence of larger and more robust data sets, a less prescriptive strategy for valve sizing in bicuspid anatomy has been advocated.^58^ This “modified annular sizing” approach incorporates annular, left ventricular outflow tract, and intercommissural distance measurements, while further adjusting for the qualitative burden of leaflet calcification. Rather than adhering to a fixed algorithm, this strategy allows for individualized adjustment, reflecting a pragmatic, experience-based synthesis of annular and supra-annular principles to better accommodate the anatomical variability of BAV. The heterogeneous aortic valvular complex anatomy and leaflet morphology of BAV patients make THV sizing challenging and lend well to the concept of machine learning–based simulation software to assist in this process. Such tools have yielded promising initial results but require robust validation in large series.^58^, ^59^, ^60^

THV undersizing—which may be employed in BAV patients with excessive leaflet calcification and/or a long calcified raphe to mitigate the risk of aortic annulus rupture or severe underexpansion—has been associated with significantly higher rates of moderate-to-severe structural valve deterioration (hazard ratio [HR] 3.05, log-rank *p* < 0.001), severe bioprosthetic valve dysfunction (HR: 2.07; log-rank *p* = 0.003), and bioprosthetic valve failure (HR: 3.25; log-rank *p* = 0.002) at 5 years in a large multicenter registry of low surgical-risk patients with BAV stenosis.^61^ Both balloon-expandable (BE) and self-expanding (SE) THV may exhibit noncircular or asymmetric expansion and eccentricity in BAV, leading to more frequent stent frame underexpansion, which is associated with an increased risk of thrombosis and other adverse cardiovascular events.^52^^,^^62^, ^63^, ^64^ Despite earlier concerns, long-term data from the STABILITY (bicuSpid TAVI duraBILITY) registry showed a 4-year all-cause mortality of 32% and only 4% moderate-to-severe valve dysfunction in intermediate-risk BAV patients,^65^ while a large multicenter registry of low surgical-risk patients reported 5-year rates of moderate-to-severe valve dysfunction and bioprosthetic valve failure of 8 and 6%, respectively—the longest follow-up available to date.^61^

## Procedural Challenges in Bicuspid Aortic Valve Disease

In non-raphe-type BAV morphologies, the absence of cusp fusion and a third commissure makes it difficult to identify a reliable implantation projection. The symmetric 2-cusp anatomy often results in an elliptical orifice and variable annular orientation, increasing the risk of parallax during THV deployment.^49^ This may lead to malpositioning, and careful fluoroscopic angle adjustment is essential to achieve coaxial alignment and accurate valve placement. TAVI should therefore be employed in well-selected patients with this anatomic variant.

Crossing the aortic valve can be technically more challenging in BAV anatomies due to the presence of a raphe (partial or complete fused BAV) or 2 dominant cusps (2-sinus BAV).^49^ In cases with severe leaflet or raphe calcification, advancing the THV delivery system may be difficult, increasing the risk of aortic injury and embolization. Use of flexible delivery systems, snare catheters, or buddy balloons can aid annular alignment and facilitate valve crossing.^49^ In heavily calcified anatomies or in case of THV mismatch, device embolization may occur, and balloon predilatation is advised to mitigate these risks.^49^

## Aortic Valve Replacement for Bicuspid Aortic Valve Stenosis—Observational Studies

### Surgical Aortic Valve Replacement

Isolated SAVR in patients with BAV is associated with favorable early and long-term outcomes in large observational registries (Supplementary Table 1 summarizes outcomes of SAVR in BAV vs. TAV). Recent data indicate that BAV patients undergoing isolated SAVR—typically around 60 years of age—exhibit early postoperative mortality rates below 2%, with a 9-year all-cause mortality of approximately 11%.^66^, ^67^, ^68^, ^69^, ^70^ Despite the increased anatomical complexity inherent to BAV morphology, multiple single-center and multicenter comparative studies have reported equivalent short-term and superior long-term outcomes in BAV patients compared to those with TAV after isolated SAVR, with significantly improved survival observed at both 5-year^71^, ^72^, ^73^ and 10-year^74^, ^75^, ^76^ follow-up when cohorts are matched for age and baseline comorbidities.

Patients with BAV requiring SAVR and concomitant ascending aortic surgery tend to be younger and present with fewer cardiovascular risk factors.^75^ Outcomes following combined aortic valve and aortic procedures are favorable, with retrospective analyses reporting 30-day mortality rates below 2% (Supplementary Table 2 summarizes outcomes of SAVR and concomitant ascending aortic surgery in BAV patients).^66^^,^^77^ Although long-term outcome data are predominantly derived from high-volume centers, reported 5-year survival rates remain high, ranging from 90% to 95% (Supplementary Table 2).^67^^,^^70^^,^^78^^,^^79^ Importantly, postoperative aortic complications and progressive aortic dilation occur more frequently in patients presenting with preoperative aortic regurgitation compared to those with AS, highlighting the need for tailored surveillance strategies in this subgroup.^80^

Although current guidelines advise considering mechanical valves for younger patients (<60 years per 2025 ESC/EACTS –class IIa, level of evidence [LoE] C; <50 years per 2020 ACC/AHA–Class IIa, LoE B-Randomized) and bioprostheses for those >65 years (Class IIa, LoE B-Randomized), routine clinical practice increasingly shows a shift to bioprostheses.^13^^,^^14^ Among 109,842 patients undergoing isolated SAVR in the Society of Thoracic Surgeons (STS)-Adult Cardiac Surgery Database registry from 2008 to 2019, the use of mechanical prostheses declined from 20% to <10%, despite evidence of lower risk-adjusted mortality in patients ≤60 years who receive mechanical valves.^81^ In the STS registry (2011-2018), mechanical valves were used in only 2.6% of BAV patients (n = 9131) undergoing SAVR.^73^ Although BAV anatomy does not inherently alter prosthesis choice, younger patients may benefit from the durability of mechanical valves.

### Transcatheter Aortic Valve Implantation

TAVI in BAV anatomy was initially limited to patients with severe AS who were inoperable or at intermediate-to-high surgical risk. Early retrospective studies among BAV patients undergoing TAVI reported lower device success and increased risks of adverse events, including aortic root injury, surgical conversion, moderate-to-severe paravalvular leak (PVL), periprocedural stroke, and all-cause mortality compared to TAV patients (Graphical Abstract; Supplementary Table 3 summarizes outcomes of TAVI in BAV stenosis, and Supplementary Table 4 summarizes comparative outcomes of TAVI in BAV vs. TAV).^62^^,^^82^, ^83^, ^84^, ^85^

These early outcomes improved over time, owing to improved procedural planning, refined techniques (Graphical Abstract and Supplementary Tables 3 and 4), and newer-generation devices (Supplementary Table 5 summarizes comparative outcomes of TAVI in BAV patients according to different types and generations of THV).^52^^,^^65^^,^^86^, ^87^, ^88^, ^89^, ^90^, ^91^ In a propensity score (PS)-matched analysis of 2691 pairs derived from the STS-ACC Transcatheter Valve Therapy (TVT) Registry, 30-day mortality was similar among BAV and TAV patients treated with BE THVs, despite higher but low absolute rates of annular rupture, surgical conversion, stroke, and pacemaker implantation in the BAV group.^92^ Similarly, the Bicuspid AS Transcatheter Aortic Valve Replacement (TAVR) study of 546 matched pairs showed no significant difference in 2-year mortality (17.2 vs. 19.4%; *p* = 0.28).^93^ Among patients undergoing TAVI with SE THVs, data from the STS-ACC TVT Registry reported comparable rates of mortality, stroke, rehospitalization, and valve performance at 1 year, but a higher rate of reintervention in BAV compared with TAV patients (1.7 vs. 0.3%; *p* = 0.01).^94^

The selection of BAV patients undergoing TAVI has been significantly refined by means of CT-based analyses, allowing for better identification of anatomies at risk of complications (Supplementary Table 6 summarizes comparative outcomes of TAVI according to different BAV phenotypes). CT-based studies in *Sievers* type 0 and type 1 BAV patients suggest that calcification patterns—more than valve morphology alone—are key determinants of outcomes. Specifically, patients with both significant calcified raphe and excessive leaflet calcification have been reported to experience higher rates of aortic root injury, PVL, and all-cause mortality at 3 years compared to those without or only one high-risk feature.^64^^,^^95^ Contemporary registry data suggest clear progress in rates of major adverse cardiovascular events (MACE), quality of life (QoL), and valve performance among carefully selected low surgical-risk BAV patients (Graphical Abstract and Supplementary Tables 3 and 4). In the low-risk TAVR and Evolut Low-Risk Bicuspid trials (n = 145 matched pairs), 1-year outcomes were comparable between BAV and TAV in terms of all-cause death, stroke, reintervention, and valve hemodynamics.^96^ These findings were supported by data up to 3 years showing sustained large improvements in QoL.^88^^,^^89^ Similarly, the Placement of AoRtic TraNscathetER valves 3 Bicuspid registry (n = 148 matched pairs) reported no difference in the composite primary endpoint (all-cause mortality, stroke, and cardiovascular rehospitalization: 10.9 vs. 10.2%; *p* = 0.80) or individual endpoints at 1 year.^97^ The most recent STS-ACC TVT analysis of 3168 matched BAV and TAV low surgical-risk patients treated with BE THVs showed no significant difference at 1 year in all-cause death, stroke, PVL, valve hemodynamics, or QoL scores.^98^

Long-term registry data indicate that BAV patients experience rates of MACE comparable to those of TAV patients undergoing TAVI, although outcomes vary across BAV subtypes. Five-year all-cause mortality appears lower in BAV (26.2%) than in TAV (45.5%; *p* < 0.0001), with pronounced differences between *Sievers* type 0 (11.0%) and type 1 (34.5%; HR: 2.38; 95% CI: 1.32–4.28; *p* = 0.004).^99^ While these findings are adjusted for known covariates, the observed differences may still partly reflect residual confounding arising from baseline variations in comorbidity burden and age, particularly the younger profile of type 0 BAV patients.^99^ In a recent multicenter study of 972 patients with raphe-type BAV stenosis, high residual gradients (≥20 mmHg) occurred in ∼4% of cases after TAVI and were independently predicted by small (≤23 mm) THV size, being associated with a more than twofold increase in 3-year MACE risk and a higher incidence of neurologic events.^100^ In BAV patients, SE THVs were associated with lower 5-year mortality than BE THVs (23.6 vs. 41.7%; HR: 1.63; 95% CI: 1.05-2.51; *p* = 0.028).^99^

### TAVI vs. SAVR in Bicuspid Aortic Valve

Current guidelines recommend SAVR as the treatment of choice in patients with BAV.^13^^,^^14^^,^^101^ Surgery is particularly preferred in patients with anatomical features that confer high risk for TAVI and those with significant aortopathy. Notwithstanding, several observational studies have reported favorable TAVI outcomes in carefully selected BAV patients compared to SAVR (Supplementary Table 7 summarizes comparative outcomes of TAVI vs. SAVR in BAV).

An inverse probability-weighted (IPW) analysis of Medicare and Medicaid data (n = 11,289) demonstrated lower in-hospital mortality with TAVI compared to SAVR (HR: 0.75; *p* = 0.038), but higher 4-year risks of all-cause mortality (HR: 1.49; *p* < 0.001), stroke (HR: 1.35; *p* < 0.001), and 5-year rehospitalization (HR: 1.23; *p* = 0.045), with these differences being more pronounced among younger, low surgical-risk patients (<75 years, STS <4%).^102^ In contrast, another IPW study without long-term follow-up (n = 56,331) reported no significant differences in early outcomes, including all-cause mortality and stroke.^103^

Propensity Score (PS)-matched analyses using large U.S. national databases have shown comparable mid-term outcomes between TAVI and SAVR in BAV patients with similar rates of all-cause mortality and stroke.^104^, ^105^, ^106^, ^107^ However, PS-matched data on short-term outcomes remain controversial. Some PS studies demonstrate similar survival rates^106^^,^^107^ or even a short-term advantage for TAVI up to 6 months.^105^; however, beyond 6 months, the most recent U.S. Medicare and Medicaid data indicate an increase in all-cause mortality (HR: 2.16; *p* = 0.008) and heart failure-related rehospitalization (HR: 4.78; *p* < 0.001) among patients undergoing TAVI compared with SAVR, reflecting possible different hazard risk profiles between these 2 treatment strategies.^104^

A meta-analysis based on nonrandomized studies encompassing 6550 patients with BAV demonstrated comparable risks of in-hospital mortality and stroke between TAVI and SAVR.^108^ However, TAVI was associated with higher rates of permanent pacemaker implantation and PVL, whereas SAVR was associated with increased rates of bleeding and acute kidney injury.^76^ A separate meta-analysis of nonrandomized studies indicated a time-dependent rise in all-cause mortality following TAVI beyond 10 months (HR: 1.7; *p* = 0.02).^109^

The recently published NOTION-2 trial compared TAVI and SAVR in low surgical-risk patients (STS score ≤4%) aged ≤75 years with severe AS (n = 370; 194 TAVI vs. 176 SAVR; mean age 71.1 years; median STS risk score 1.1%), including individuals with both TAV (n = 270) and BAV (n = 100) anatomies.^34^^,^^110^ At 3-year follow-up, the composite primary endpoint (all-cause mortality, stroke, or procedure-, valve-, or heart failure-related rehospitalization) occurred in 16.1% of patients in the TAVI group and 12.6% in the SAVR group (HR: 1.3; 95% CI: 0.8 – 2.2; *p* = 0.4). While outcomes were comparable between TAVI and SAVR in the TAV subgroup, a numerically higher event rate favoring SAVR was observed in the BAV subgroup at 1 and 3 years (20.4 vs. 7.8%; absolute risk difference: 12.6%; 95% CI: –0.1 to 26.0%; HR: 2.8; 95% CI: 0.9 – 9.0; *p* = 0.08) (Graphical Abstract). These findings should be interpreted with caution, given the limited sample size, insufficient statistical power to confirm the observed differences, imprecise risk estimates, and the relatively short follow-up period of only 3 years. The UK TAVI trial compared TAVI and SAVR in low surgical-risk patients aged ≥75 years with severe AS (n = 913; 498 TAVI vs. 455 SAVR; median age 81 years; median STS risk score 2.6%, including individuals with both TAV and BAV anatomies although the distribution of each of the 2 anatomies was not reported).^111^ Among patients with BAV anatomy, the rate of stroke was 16% at 5 years compared with 13% among those with TAV (unpublished data, presented at the EuroPCR 2025 congress). Importantly, these 2 trials did not apply morphological BAV exclusion criteria or utilize imaging-based stratification for BAV anatomy or calcification burden; arguably, the NOTION-2 trial also did not reflect contemporary “best practice” for TAVI in BAV, treating some cases of unicuspid (*Sievers* type 2) BAV morphology (data on the type of BAV not available for the UK TAVI trial).

## Upcoming Randomized Controlled Trials

It is widely agreed that assessing the comparative safety and effectiveness of TAVI and SAVR in patients with BAV stenosis is essential to address an important evidence gap and to provide high-quality data to inform guidelines, particularly due to the higher rate of BAV stenosis in younger patients and the global trend of expansion of TAVI to younger and lower-risk patients with longer life expectancies.^12^^,^^35^^,^^37^^,^^86^^,^^104^^,^^112^

### Trials Design

**Figure 2:**
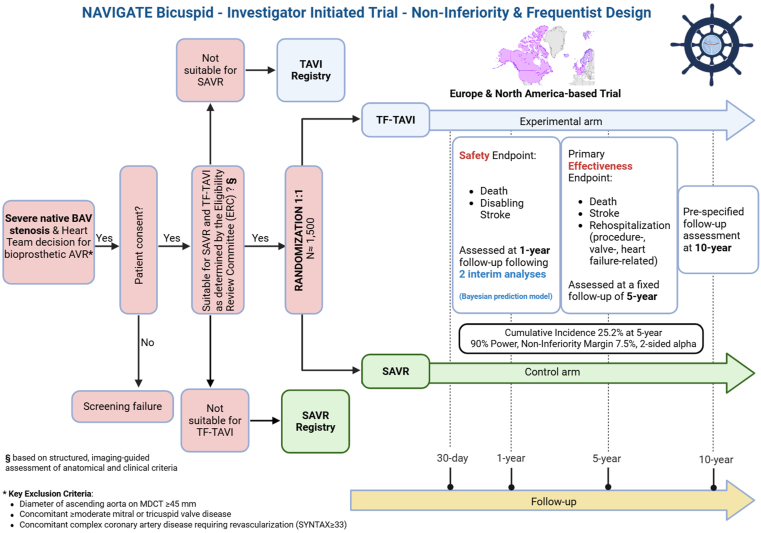
**Design of the upcoming NAVIGATE Bicuspid trial.** Abbreviations: AVR, aortic valve replacement; BAV, bicuspid aortic valve; ERC, Eligibility Review Committee; MDCT, multidetector computed tomography; SAVR, surgical aortic valve replacement; TAVI, transcatheter aortic valve implantation; TF, transfemoral.

**Figure 3:**
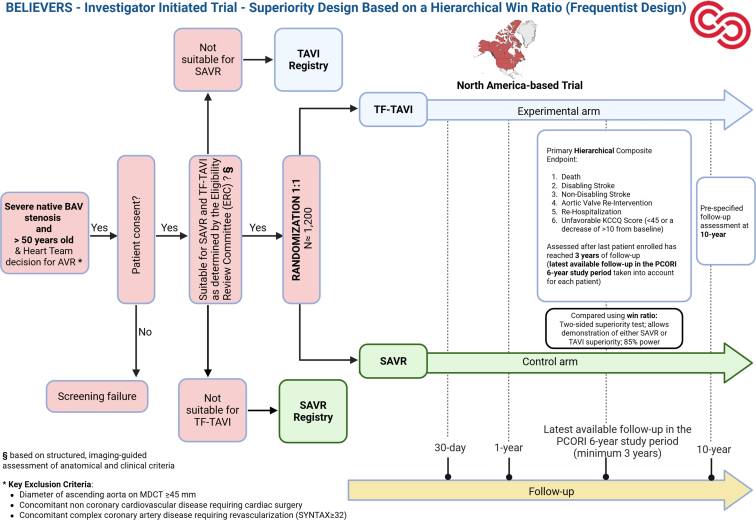
**Design of the upcoming BELIEVERS trial.** Abbreviations: AVR, aortic valve replacement; BAV, bicuspid aortic valve; ERC, Eligibility Review Committee; KCCQ, Kansas City Cardiomyopathy Questionnaire; MDCT, multidetector computed tomography; PCORI, Patient-Centered Outcomes Research Institute; SAVR, surgical aortic valve replacement; TAVI, transcatheter aortic valve implantation; TF, transfemoral.

Two investigator-initiated RCTs—the TraNscatheter Aortic Valve Implantation versus surGical AorTic valvE replacement in patients with Bicuspid aortic valve stenosis (NAVIGATE Bicuspid) (Figure 2 and Graphical Abstract) and Bicuspid aortic valve replacement: EvaLuatIon of transcathetEr VERsus Surgery (BELIEVERS) trials (Figure 3 and Graphical Abstract)—are currently in preparation to compare transfemoral TAVI with SAVR in patients with severe native BAV stenosis. The NAVIGATE Bicuspid trial is endorsed by the Global Cardiovascular Research Funders Forum (GCRFF), and the BELIEVERS trial is endorsed by the Patient-Centered Outcomes Research Institute. Patient eligibility for a bioprosthetic aortic valve replacement and their suitability for both procedures (transfemoral TAVI and SAVR) will be confirmed by a central eligibility review committee based on a comprehensive clinical and imaging assessment (see Figures 2 and 3 for key exclusion criteria such as an ascending aorta diameter ≥45 mm).

The BELIEVERS trial is a superiority study, designed with a hierarchical win ratio as the primary analytic approach. Superiority testing is two-sided, permitting demonstration of superiority for either SAVR or TAVI. The NAVIGATE Bicuspid trial is a noninferiority study, assuming a 5-year cumulative incidence of the primary effectiveness composite endpoint of 25.2% in both groups and applying a noninferiority margin of 7.5% for the between-group difference. If TAVI is demonstrated to be noninferior to SAVR with respect to the primary composite effectiveness endpoint at a 2-sided *p* value <0.05, subsequent testing for superiority will be conducted. These trials are designed to generate robust, adequately powered estimates of the comparative safety and effectiveness of both interventions over extended follow-up periods.

Alongside RCTs, companion registries for TAVI and SAVR in BAV patients will be established to assess the external validity of the trial populations, offering insight into differences between trial participants and patients treated in routine clinical practice.

The NAVIGATE and BELIEVERS investigators will use bioprostheses with Food and Drug Administration approval or “Conformité Européenne” mark for use in BAV anatomy during the conduct of the trial. The choice of bioprosthesis will be left at the operator/Heart Team's discretion, and prespecified subgroup analyses according to the type of TAVI devices implanted (BE vs. SE) will be performed.

### Endpoint Selection

The primary endpoint proposed in the BELIEVERS trial is a hierarchical composite patient-centered outcome (assessed using the win ratio) of (i) all-cause death, (ii) disabling stroke, (iii) nondisabling stroke, (iv) aortic valve reintervention, (v) rehospitalization, (vi) unfavorable Kansas City Cardiomyopathy Questionnaire (KCCQ) score (<45 or a decrease of >10 from baseline) as per Valve Academic Research Consortium 3 (VARC-3)^113^ assessed at the latest available follow-up within the funded 6-year study period, and the primary effectiveness endpoint proposed in the NAVIGATE Bicuspid trial is a composite of all-cause death, any stroke, or re-hospitalization (procedure-, valve-, or heart failure-related) (VARC-3 definitions)^113^ assessed at a fixed follow-up of 5 years. A key distinction between the 2 trials is the substantially longer follow-up period for assessing the primary endpoint. BELIEVERS and NAVIGATE Bicuspid will evaluate primary outcomes at 5 years and beyond, in contrast to the 1-year follow-up used in earlier trials. This long-term follow-up will provide critical insights into the long-term safety and effectiveness of TAVI compared to SAVR in patients with BAV. The primary composite endpoint in both upcoming BAV trials will also be assessed up to 10 years. The NAVIGATE Bicuspid trial includes a safety composite endpoint of all-cause death or disabling stroke at 1 year, with 2 interim analyses to detect early harm of TAVI at 1 year. In the BELIEVERS trial, the key secondary outcome is a hierarchical composite of (i) all-cause death and (ii) KCCQ score (compared using the win ratio), at the latest available follow-up.

Of note, the use of harmonized endpoint definitions according to VARC-3 will enable pooled analyses. Importantly, current guidelines require conclusive evidence—usually from at least 2 adequately powered RCTs free of major bias, with substantial evidence against the play of chance when combined in a meta-analysis—to qualify for a LoE A.^114^

### Cerebral Embolic Protection Devices

BAV stenosis patients are known to have more extensive calcification than TAV, and emboli risk therefore may be a concern. Although cerebral embolic protection (CEP) devices effectively reduce embolic burden,^115^^,^^116^ they have failed to show clinical benefit in large-scale clinical trials^117^^,^^118^ and neurocognitive benefits remain inconclusive.^119^^,^^120^ Of note, only a minority of patients included in the trials investigating the use of CEP devices had native BAV (∼8.5% in the Stroke PROTECTion with SEntinel During Transcatheter Aortic Valve Replacement [PROTECTED TAVR]^117^ and British Heart Foundation Randomised Trial of Routine Cerebral Embolic Protection in Transcatheter Aortic Valve Implantation (BHF PROTECT-TAVI)^118^ trials). Notably, in the subgroup of patients with BAV anatomy enrolled in the randomized NOTION-2 trial, the proportion of stroke (1-year: 6.1 vs. 0%, *p* = 0.07; 3-year absolute risk difference: 6.2%, 95% CI −2.4 to 14.8) and disabling stroke (1-year: 2.0 vs. 0%, *p* = 0.30; 3-year absolute risk difference: 2.0%, 95% CI −1.9 to 6.0) was numerically higher following TAVI compared to SAVR.^34^ However, these differences were not statistically significant, and risk estimates were imprecise.^34^ Furthermore, the prospective Transcatheter aORtic valve replacement single center registry in CHinese population (TORCH) study demonstrated that, compared with TAV, BAV patients had a higher number of early post-TAVI new lesions, total lesion volume, and volume per lesion in diffusion-weighted imaging magnetic resonance imaging.^121^ Therefore, selective use of CEP devices in BAV patients—particularly those with bulky, irregular calcifications—may be considered, and their use in the NAVIGATE bicuspid and BELIEVERS trials will be left to the discretion of operators. A dedicated cerebral magnetic resonance imaging substudy embedded within the NAVIGATE Bicuspid trial, incorporating mid-term functional and neurocognitive assessments, will provide important insights into this patient population and help elucidate the potential differential effects of TAVI vs. SAVR on cerebral outcomes in individuals with BAV.

### Concomitant Coronary Artery Disease

Both the NAVIGATE Bicuspid and BELIEVERS trials will allow for inclusion of patients with coronary artery disease (CAD) requiring revascularization (coronary artery bypass grafting [CABG] if SAVR arm vs. percutaneous coronary intervention (PCI) if TAVI arm) to ensure broader generalizability and reflect real-world clinical practice. In prior low-to-intermediate surgical risk trials comparing TAVI and SAVR in TAV patients, the proportion of patients with CAD undergoing concomitant revascularization at the time of aortic valve replacement ranged from 3.9 to 14.5% for PCI in case of TAVI and from 12.8 to 22.1% for CABG in case of SAVR.^22^, ^23^, ^24^, ^25^^,^^122^ Similarly, data from the Nationwide FinnValve registry indicate that among patients with BAV stenosis, concomitant PCI was performed in 6.0% of TAVI patients and concomitant CABG in 27.9% of SAVR patients.^123^ The presence of CAD is unlikely to bias the results of the NAVIGATE Bicuspid and BELIEVERS trials, as complex CAD (left main disease including the bifurcation, complex multivessel CAD with high SYNTAX score) will be excluded, randomization will be stratified/minimized by planned revascularization strategy (as determined by the Heart Team), and standardized guidance^13^^,^^14^ will ensure consistent lesion assessment and revascularization decisions.

### Patient and Public Involvement

Embedding patients’ perspectives in the trial designs and establishing mechanisms for a continuous representation of the patients’ voice throughout these trials is important to ensure results are relevant to the patient population under investigation.^124^^,^^125^ The trial objectives receive support from the national cardiology societies and a dedicated heart valve charity, and BELIEVERS has been endorsed by the Patient-Centered Outcomes Research Institute. Patient and Public Involvement Officers will coordinate and supervise the implementation of Patient and Public Involvement activities for the duration of both trials. Dedicated Patient Advisory Groups, composed of individuals with lived BAV disease—whether having undergone TAVI, SAVR, or being intervention-naive—as well as care providers, will provide ongoing input to ensure that patient perspectives inform trial processes and decision-making. In addition, patient-reported outcomes —including New York Heart Association (NYHA) functional class—and QoL, assessed using the KCCQ score, are integral components of the trials’ endpoints. These findings will provide valuable insights for patients regarding functional status and QoL and will also inform health care resource planning.

## Conclusions

SAVR remains the current standard of care for severe BAV stenosis in low surgical-risk patients. TAVI represents a less invasive treatment alternative, with registry data suggesting comparable short- and mid-term outcomes to SAVR in appropriately selected BAV patients. Due to the unique anatomical and procedural challenges, BAV patients are best managed in specialized Heart Valve Centers. To date, all RCTs comparing TAVI and SAVR have excluded BAV patients, except the UK TAVI trial and the recent NOTION-2 trial—the latter suggesting higher 3-year event rates of the composite of all-cause mortality, stroke, and rehospitalization in low surgical-risk BAV patients treated with transfemoral TAVI compared to those receiving SAVR. The planned NAVIGATE Bicuspid and BELIEVERS trials will directly compare TAVI and SAVR in BAV populations. These trials are designed to provide robust, adequately powered estimates of the comparative safety and effectiveness of both interventions over extended follow-up and are expected to inform future guideline recommendations in the setting of BAV anatomy. The trials will provide valuable insights for patients regarding functional status and QoL and will also inform health care resource planning.

## Ethics Statement

Not applicable to this review.

## Funding

The authors have no funding to report.

## Disclosure Statement

S. Windecker reports research and educational grants to the institution from Abbott, Amgen, AstraZeneca, BMS, Bayer, Biotronik, Boston Scientific, Cardinal Health, CardioValve, CSL Behring, Daiichi Sankyo, Edwards Lifesciences, Guerbet, InfraRedx, Johnson & Johnson, Medicure, Medtronic, Novartis, Polares, OrPha Suisse, Pfizer, Regeneron, Sanofi-Aventis, Sinomed, Terumo, and V-Wave; serves as an unpaid advisory board member and/or unpaid member of the steering/executive group of trials funded by Abbott, Abiomed, Amgen, AstraZeneca, BMS, Boston Scientific, Biotronik, Cardiovalve, Edwards Lifesciences, Med Alliance, Medtronic, Novartis, Polares, Sinomed, V-Wave, and Xeltis, but has not received personal payments by pharmaceutical companies or device manufacturers; is also a member of the steering/executive committee group of several investigator-initiated trials that receive funding by industry without impact on his personal remuneration; is an unpaid member of the Pfizer Research Award selection committee in Switzerland and of the Women as One Awards Committee; is a member of the Clinical Study Group of the Deutsches Zentrum für Herz Kreislauf-Forschung and of the Advisory Board of the Australian Victorian Heart Institute; and is the chairperson of the ESC Congress Program Committee and Deputy Editor of JACC Cardiovascular Interventions. P. Jüni serves as an unpaid member of the steering group of trials funded by AstraZeneca, Biotronik, Biosensors, St Jude Medical, and The Medicines Company, and received research grants to their institution from AstraZeneca, Biotronik, Biosensors International, Eli Lilly, and The Medicines Company, and honoraria to their institution for participation in advisory boards from Amgen, but has not received personal payments by any pharmaceutical company or device manufacturer; and is a Tier 1 Canada Research Chair in Clinical Epidemiology of Chronic Diseases. This research was completed, in part, with funding from the Canada Research Chairs Programme. R.R. Makkar reports research grant support from Boston Scientific, Edwards Lifesciences, Medtronic, Abbott, JenaValve, and Protembis. H. Jilaihawi has received institutional grants for clinical research in Pi-Cardia; holds equity and serves on the scientific advisory board for DASI Simulations; and has served as a consultant to Edwards Lifesciences and Medtronic. A. Gupta has received grant support from National Heart, Lung, and Blood Institute 1R56HL175516-01; and is a co-founder of Heartbeat Health, Inc, a telehealth cardiology company, and iCardio.ai, an artificial intelligence echocardiography company. M.A. Borger discloses that his hospital receives speakers’ honoraria and/or consulting fees on his behalf from Edwards Lifesciences, Medtronic, Abbott, and Artivion. M.B. Leon has received grant from Edwards Lifesciences, Abbott Vascular, and Medtronic, consultant fees from Foldax and Anteris, is in data safety monitoring board with Medtronic, have leadership role in Heart Valve Collaboratory and stocks in Pi-Cardia. D. Samim received funding for an online course from Edwards Lifesciences and received institutional funding for an online course from Edwards Lifesciences, but no personal payments.

The other author had no conflicts to declare.

## References

[1] Iung B., Delgado V., Rosenhek R. (2019). Contemporary presentation and management of valvular heart disease: the EURObservational research programme valvular heart disease II survey. Circulation.

[2] Yadgir S., Johnson C.O., Aboyans V. (2020). Global, regional, and national burden of calcific aortic valve and degenerative mitral valve diseases, 1990-2017. Circulation.

[3] Cribier A., Eltchaninoff H., Bash A. (2002). Percutaneous transcatheter implantation of an aortic valve prosthesis for calcific aortic stenosis: first human case description. Circulation.

[4] Ryffel C., Windecker S., Pilgrim T. (2023). Expansion of transcatheter aortic valve implantation and mortality due to aortic stenosis between 2010 and 2019. Circ Cardiovasc Interv.

[5] Otto C.M. (2002). Calcification of bicuspid aortic valves. Heart.

[6] Houessou U., Zamani P., Manikpurage H.D. (2025). Transcriptomic signatures of calcific aortic valve stenosis severity in human tricuspid and bicuspid aortic valves. JACC Basic Translational Sci.

[7] Roberts W.C., Ko J.M. (2005). Frequency by decades of unicuspid, bicuspid, and tricuspid aortic valves in adults having isolated aortic valve replacement for aortic stenosis, with or without associated aortic regurgitation. Circulation.

[8] Yang L.T., Ye Z., Wajih Ullah M. (2023). Bicuspid aortic valve: long-term morbidity and mortality. Eur Heart J.

[9] Pujari S.H., Agasthi P. (2023). Statpearls.

[10] Coffey S., Cairns B.J., Iung B. (2016). The modern epidemiology of heart valve disease. Heart.

[11] Masri A., Svensson L.G., Griffin B.P., Desai M.Y. (2017). Contemporary natural history of bicuspid aortic valve disease: a systematic review. Heart.

[12] Mehta C.K., Liu T.X., Bonnell L. (2024). Age-stratified surgical aortic valve replacement for aortic stenosis. Ann Thorac Surg.

[13] Otto C.M., Nishimura R.A., Bonow R.O. (2021). 2020 ACC/AHA guideline for the management of patients with valvular heart disease: executive summary: a report of the American college of cardiology/American heart association joint committee on clinical practice guidelines. Circulation.

[14] Praz F., Borger M.A., Lanz J. (2025). 2025 ESC/EACTS guidelines for the management of valvular heart disease. Eur Heart J.

[15] ESC Total annual number of TAVI procedures performed in adult patients (per million people) with aortic stenosis/regurgitation in Europe 2023. https://eatlas.escardio.org/Data/Cardiovascular-healthcare-delivery/Interventional-cardiology-procedures/sipcp_ptavi_1m_r-percutaneous-aortic-valve-implantation-tavi-per-million-pe.

[16] Pilgrim T., Maznyczka A. (2024). Transcatheter valves for bicuspid aortic stenosis: navigating anatomical challenges and tackling a cocktail of risks. J Am Coll Cardiol Intv.

[17] Kim W.K., Liebetrau C., Fischer-Rasokat U. (2020). Challenges of recognizing bicuspid aortic valve in elderly patients undergoing TAVR. Int J Cardiovasc Imaging.

[18] Gupta T., DeVries J.T., Gilani F., Hassan A., Ross C.S., Dauerman H.L. (2024). Temporal trends in transcatheter aortic valve replacement for isolated severe aortic stenosis. J Soc Cardiovasc Angiography Interv.

[19] Adams D.H., Popma J.J., Reardon M.J. (2014). Transcatheter aortic-valve replacement with a self-expanding prosthesis. N Engl J Med.

[20] Gleason T.G., Reardon M.J., Popma J.J. (2018). 5-Year outcomes of self-expanding transcatheter versus surgical aortic valve replacement in high-risk patients. J Am Coll Cardiol.

[21] Leon M.B., Smith C.R., Mack M. (2010). Transcatheter aortic-valve implantation for aortic stenosis in patients who cannot undergo surgery. N Engl J Med.

[22] Leon M.B., Smith C.R., Mack M.J. (2016). Transcatheter or surgical aortic-valve replacement in intermediate-risk patients. N Engl J Med.

[23] Mack M.J., Leon M.B., Thourani V.H. (2019). Transcatheter aortic-valve replacement with a balloon-expandable valve in low-risk patients. N Engl J Med.

[24] Popma J.J., Deeb G.M., Yakubov S.J. (2019). Transcatheter aortic-valve replacement with a self-expanding valve in low-risk patients. N Engl J Med.

[25] Reardon M.J., Van Mieghem N.M., Popma J.J. (2017). Surgical or transcatheter aortic-valve replacement in intermediate-risk patients. N Engl J Med.

[26] Van Mieghem N.M., Deeb G.M., Sondergaard L. (2022). Self-expanding transcatheter vs surgical aortic valve replacement in intermediate-risk patients: 5-year outcomes of the SURTAVI randomized clinical trial. JAMA Cardiol.

[27] Jorgensen T.H., Thyregod H.G.H., Ihlemann N. (2021). Eight-year outcomes for patients with aortic valve stenosis at low surgical risk randomized to transcatheter vs. surgical aortic valve replacement. Eur Heart J.

[28] Leon M.B., Mack M.J., Hahn R.T. (2021). Outcomes 2 years after transcatheter aortic valve replacement in patients at low surgical risk. J Am Coll Cardiol.

[29] Forrest J.K., Deeb G.M., Yakubov S.J. (2023). 3-Year outcomes after transcatheter or surgical aortic valve replacement in low-risk patients with aortic stenosis. J Am Coll Cardiol.

[30] Mack M.J., Leon M.B., Thourani V.H. (2023). Transcatheter aortic-valve replacement in low-risk patients at five years. N Engl J Med.

[31] Blankenberg S., Seiffert M., Vonthein R. (2024). Transcatheter or surgical treatment of aortic-valve stenosis. N Engl J Med.

[32] Forrest J., Yakubov S., Deeb G. (2025). 5-Year outcomes after transcatheter or surgical aortic valve replacement in low-risk patients with aortic stenosis. JACC.

[33] Sharma T., Krishnan A.M., Lahoud R., Polomsky M., Dauerman H.L. (2022). National trends in TAVR and SAVR for patients with severe isolated aortic stenosis. J Am Coll Cardiol.

[34] Jørgensen T.H., Thyregod H.G.H., Savontaus M. (2024). Transcatheter aortic valve implantation in low-risk tricuspid or bicuspid aortic stenosis: the NOTION-2 trial. Eur Heart J.

[35] Sievers H.H., Schmidtke C. (2007). A classification system for the bicuspid aortic valve from 304 surgical specimens. J Thorac Cardiovasc Surg.

[36] Jilaihawi H., Chen M., Webb J. (2016). A bicuspid aortic valve imaging classification for the TAVR era. JACC Cardiovasc Imaging.

[37] Michelena H.I., Della Corte A., Evangelista A. (2021). International consensus statement on nomenclature and classification of the congenital bicuspid aortic valve and its aortopathy, for clinical, surgical, interventional and research purposes. Eur J Cardiothorac Surg.

[38] Kong W.K.F., Bax J.J., Michelena H.I., Delgado V. (2020). Sex differences in bicuspid aortic valve disease. Prog Cardiovasc Dis.

[39] Jilaihawi H., Wu Y., Yang Y. (2015). Morphological characteristics of severe aortic stenosis in China: imaging corelab observations from the first Chinese transcatheter aortic valve trial. Catheter Cardiovasc Interv.

[40] Rodríguez-Palomares J.F., Dux-Santoy L., Guala A., Galian-Gay L., Evangelista A. (2023). Mechanisms of aortic dilation in patients with bicuspid aortic valve: JACC state-of-the-art review. J Am Coll Cardiol.

[41] Michelena H.I., Della Corte A., Prakash S.K., Milewicz D.M., Evangelista A., Enriquez-Sarano M. (2015). Bicuspid aortic valve aortopathy in adults: incidence, etiology, and clinical significance. Int J Cardiol.

[42] Harris K.M., Nienaber C.A., Peterson M.D. (2022). Early mortality in type A acute aortic dissection: insights from the international registry of acute aortic dissection. JAMA Cardiol.

[43] Sillesen A.S., Vøgg O., Pihl C. (2021). Prevalence of bicuspid aortic valve and associated aortopathy in newborns in copenhagen, Denmark. Jama.

[44] Borger M.A., Fedak P.W.M., Stephens E.H. (2018). The American association for thoracic surgery consensus guidelines on bicuspid aortic valve-related aortopathy: full online-only version. J Thorac Cardiovasc Surg.

[45] Zheng H.J., Cheng Y.B., Lin D.Q. (2023). Effect of transcatheter aortic valve replacement on ascending aorta dilatation rate in patients with tricuspid and bicuspid aortic stenosis. Int J Cardiol Heart Vasc.

[46] Jia Y., Tirado-Conte G., Montarello N. (2023). Prognostic impact of ascending aortic dilatation in bicuspid TAVR patients. JACC Cardiovasc Interv.

[47] Fan J., Li Z., Lin D. (2024). Long-term outcomes in patients with bicuspid valve stenosis and aortic dilation undergoing transcatheter valve implantation. Int J Cardiol.

[48] Lim S.M., Ahn J.M., Kang D.Y. (2024). Volume-adjusted annular sizing of balloon-expandable transcatheter heart valves for severe bicuspid aortic valve stenosis. JACC Cardiovasc Interv.

[49] Barbanti M., Costa G., Windecker S. (2025). Bicuspid aortic valve disease: advancements and challenges of transcatheter aortic valve implantation. Eur Heart J.

[50] Moharem-Elgamal S., Yeong M., Veerappan S. (2021). Feasibility and effectiveness of transcatheter aortic valve implantation in adults with congenital heart disease. Int J Cardiol Congenit Heart Dis.

[51] Sheth T., Ismail U., Chavarria J. (2025). The ABC bicuspid sizing protocol for SAPIEN 3 balloon-expandable valves. Struct Heart.

[52] Tchetche D., de Biase C., van Gils L. (2019). Bicuspid aortic valve anatomy and relationship with devices: the BAVARD multicenter registry. Circ Cardiovasc Interv.

[53] Blackman D., Gabbieri D., Del Blanco B.G. (2021). Expert consensus on sizing and positioning of SAPIEN 3/Ultra in bicuspid aortic valves. Cardiol Ther.

[54] Iannopollo G., Romano V., Buzzatti N. (2020). Supra-annular sizing of transcatheter aortic valve prostheses in raphe-type bicuspid aortic valve disease: the LIRA method. Int J Cardiol.

[55] Yao J., Wu D., Yan Y. (2025). Redefining TAVR valve sizing: a validated multiplanar approach for both bicuspid and tricuspid valves. JACC Cardiovasc Interv.

[56] Petronio A.S., Angelillis M., De Backer O. (2020). Bicuspid aortic valve sizing for transcatheter aortic valve implantation: development and validation of an algorithm based on multi-slice computed tomography. J Cardiovasc Comput Tomogr.

[57] Weir-McCall J.R., Attinger-Toller A., Blanke P. (2020). Annular versus supra-annular sizing for transcatheter aortic valve replacement in bicuspid aortic valve disease. J Cardiovasc Comput Tomogr.

[58] Jilaihawi H., Patel V., Makkar R. (2025). Sizing for transcatheter aortic valve replacement in bicuspid aortic valve anatomy. JACC Cardiovasc Interv.

[59] Yeats B.B., Sivakumar S.K., Samaee M. (2023). Calcium fracture and device over expansion in transcatheter aortic valve replacement for bicuspid aortic valves. Ann Biomed Eng.

[60] Dowling C., Bavo A.M., El Faquir N. (2019). Patient-specific computer simulation of transcatheter aortic valve replacement in bicuspid aortic valve morphology. Circ Cardiovasc Imaging.

[61] Jia Y., Maznyczka A., Boiago M. (2025). Long-term durability of transcatheter aortic valves in patients with bicuspid aortic stenosis. Catheter Cardiovasc Interv.

[62] Hayashida K., Bouvier E., Lefevre T. (2013). Transcatheter aortic valve implantation for patients with severe bicuspid aortic valve stenosis. Circ Cardiovasc Interv.

[63] Perlman G.Y., Blanke P., Dvir D. (2016). Bicuspid aortic valve stenosis: favorable early outcomes with a next-generation transcatheter heart valve in a multicenter study. JACC Cardiovasc Interv.

[64] Nagasaka T., Patel V., Shechter A. (2024). Impact of balloon-expandable TAVR valve deformation and calcium distribution on outcomes in bicuspid aortic valve. JACC Cardiovasc Interv.

[65] Fiorina C., Massussi M., Ancona M. (2023). Mid-term outcomes and hemodynamic performance of transcatheter aortic valve implantation in bicuspid aortic valve stenosis: insights from the bicuSpid TAvi duraBILITY (STABILITY) registry. Catheter Cardiovasc Interv.

[66] Svensson L.G., Kim K.H., Blackstone E.H. (2011). Bicuspid aortic valve surgery with proactive ascending aorta repair. J Thorac Cardiovasc Surg.

[67] Kaneko T., Shekar P., Ivkovic V. (2018). Should the dilated ascending aorta be repaired at the time of bicuspid aortic valve replacement?. Eur J Cardiothorac Surg.

[68] Miceli A., Berretta P., Fiore A. (2020). Sutureless and rapid deployment implantation in bicuspid aortic valve: results from the sutureless and rapid-deployment aortic valve replacement international registry. Ann Cardiothorac Surg.

[69] Glaser N., Jackson V., Eriksson P., Sartipy U., Franco-Cereceda A. (2021). Relative survival after aortic valve surgery in patients with bicuspid aortic valves. Heart.

[70] Haunschild J., Misfeld M., Schroeter T. (2020). Prevalence of permanent pacemaker implantation after conventional aortic valve replacement-a propensity-matched analysis in patients with a bicuspid or tricuspid aortic valve: a benchmark for transcatheter aortic valve replacement. Eur J Cardiothorac Surg.

[71] Huntley G.D., Thaden J.J., Alsidawi S. (2018). Comparative study of bicuspid vs. tricuspid aortic valve stenosis. Eur Heart J Cardiovasc Imaging.

[72] Holmgren A., Enger T.B., Naslund U. (2021). Long-term results after aortic valve replacement for bicuspid or tricuspid valve morphology in a Swedish population. Eur J Cardiothorac Surg.

[73] Hirji S.A., Wegermann Z., Vemulapalli S. (2023). Benchmarking outcomes of surgical aortic valve replacement in patients with bicuspid aortic valves. Ann Thorac Surg.

[74] Coti I., Werner P., Kaider A. (2022). Rapid-deployment aortic valve replacement for patients with bicuspid aortic valve: a single-centre experience. Eur J Cardiothorac Surg.

[75] Celik M., Milojevic M., Durko A.P., Oei F.B.S., Bogers A., Mahtab E.A.F. (2021). Differences in baseline characteristics and outcomes of bicuspid and tricuspid aortic valves in surgical aortic valve replacement. Eur J Cardiothorac Surg.

[76] Makkinejad A., Satija D., Monaghan K. (2025). The impact of bicuspid aortic valve on long-term outcomes after bioprosthetic valve replacement. Ann Thorac Surg.

[77] Brown B., Le T., Naeem A. (2021). Stentless valves for bicuspid and tricuspid aortic valve disease. JTCVS Open.

[78] Celik M., Mahtab E.A.F., Bogers A. (2021). Surgical aortic valve replacement with concomitant aortic surgery in patients with purely bicuspid aortic valve and associated aortopathy. J Cardiovasc Dev Dis.

[79] Rinewalt D., McCarthy P.M., Malaisrie S.C. (2014). Effect of aortic aneurysm replacement on outcomes after bicuspid aortic valve surgery: validation of contemporary guidelines. J Thorac Cardiovasc Surg.

[80] Girdauskas E., Disha K., Secknus M., Borger M., Kuntze T. (2013). Increased risk of late aortic events after isolated aortic valve replacement in patients with bicuspid aortic valve insufficiency versus stenosis. J Cardiovasc Surg (Torino).

[81] Bowdish M.E., Mehaffey J.H., Chang S.C. (2025). Bioprosthetic vs mechanical aortic valve replacement in patients 40 to 75 years of age. J Am Coll Cardiol.

[82] Mylotte D., Lefevre T., Sondergaard L. (2014). Transcatheter aortic valve replacement in bicuspid aortic valve disease. J Am Coll Cardiol.

[83] Bauer T., Linke A., Sievert H. (2014). Comparison of the effectiveness of transcatheter aortic valve implantation in patients with stenotic bicuspid versus tricuspid aortic valves (from the German TAVI registry). Am J Cardiol.

[84] Yousef A., Simard T., Webb J. (2015). Transcatheter aortic valve implantation in patients with bicuspid aortic valve: a patient level multi-center analysis. Int J Cardiol.

[85] Yoon S.H., Lefèvre T., Ahn J.M. (2016). Transcatheter aortic valve replacement with Early- and new-generation devices in bicuspid aortic valve stenosis. J Am Coll Cardiol.

[86] Halim S.A., Edwards F.H., Dai D. (2020). Outcomes of transcatheter aortic valve replacement in patients with bicuspid aortic valve disease: a report from the society of thoracic surgeons/American college of cardiology transcatheter valve therapy registry. Circulation.

[87] Tchétché D., Ziviello F., De Biase C. (2023). Transcatheter aortic valve implantation with the evolut platform for bicuspid aortic valve stenosis: the international, multicentre, prospective BIVOLUTX registry. EuroIntervention.

[88] Zahr F., Ramlawi B., Reardon M.J. (2024). 3-Year outcomes from the evolut low risk TAVR bicuspid study. JACC Cardiovasc Interv.

[89] Forrest J.K., Ramlawi B., Deeb G.M. (2021). Transcatheter aortic valve replacement in low-risk patients with bicuspid aortic valve stenosis. JAMA Cardiol.

[90] Boiago M., Bellamoli M., De Biase C. (2024). Three-year clinical outcomes after transcatheter aortic valve implantation in patients with bicuspid aortic disease: comparison between self-expanding and balloon-expandable valves. Catheter Cardiovasc Interv.

[91] Mangieri A., Tchetchè D., Kim W.K. (2020). Balloon versus self-expandable valve for the treatment of bicuspid aortic valve stenosis: insights from the BEAT International collaborative registrys. Circ Cardiovasc Interv.

[92] Makkar R.R., Yoon S.H., Leon M.B. (2019). Association between transcatheter aortic valve replacement for bicuspid vs tricuspid aortic stenosis and mortality or stroke. Jama.

[93] Yoon S.H., Bleiziffer S., De Backer O. (2017). Outcomes in transcatheter aortic valve replacement for bicuspid versus tricuspid aortic valve stenosis. J Am Coll Cardiol.

[94] Forrest J.K., Kaple R.K., Ramlawi B. (2020). Transcatheter aortic valve replacement in bicuspid versus tricuspid aortic valves from the STS/ACC TVT registry. JACC Cardiovasc Interv.

[95] Yoon S.H., Kim W.K., Dhoble A. (2020). Bicuspid aortic valve morphology and outcomes after transcatheter aortic valve replacement. J Am Coll Cardiol.

[96] Deeb G.M., Reardon M.J., Ramlawi B. (2022). Propensity-matched 1-Year outcomes following transcatheter aortic valve replacement in low-risk bicuspid and tricuspid patients. JACC Cardiovasc Interv.

[97] Williams M.R., Jilaihawi H., Makkar R. (2022). The PARTNER 3 bicuspid registry for transcatheter aortic valve replacement in low-surgical-risk patients. JACC Cardiovasc Interv.

[98] Makkar R.R., Yoon S.H., Chakravarty T. (2021). Association between transcatheter aortic valve replacement for bicuspid vs tricuspid aortic stenosis and mortality or stroke among patients at low surgical risk. JAMA.

[99] Li W., Jia Y., Li H. (2025). Long-term outcomes of BAV-0 patients compared with BAV-1 and TAV patients after TAVR. JACC Cardiovasc Interv.

[100] Tartaglia F., Gitto M., Kim W.K. (2025). High residual gradients after transcatheter aortic valve implantation in raphe-type bicuspid aortic valve stenosis: insights from the AD-HOC registry. Clin Res Cardiol.

[101] Windecker S., Okuno T., Unbehaun A., Mack M., Kapadia S., Falk V. (2022). Which patients with aortic stenosis should be referred to surgery rather than transcatheter aortic valve implantation?. Eur Heart J.

[102] Mehaffey J.H., Jagadeesan V., Kawsara M. (2025). Transcatheter vs surgical aortic valve replacement in bicuspid aortic valves. Ann Thorac Surg.

[103] Sanaiha Y., Hadaya J.E., Tran Z., Shemin R.J., Benharash P. (2023). Transcatheter and surgical aortic valve replacement in patients with bicuspid aortic valve stenosis. Ann Thorac Surg.

[104] Chen Q., Malas J., Megna D. (2024). Bicuspid aortic stenosis: national three-year outcomes of transcatheter versus surgical aortic valve replacement among medicare beneficiaries. J Thorac Cardiovasc Surg.

[105] Majmundar M., Kumar A., Doshi R. (2022). Early outcomes of transcatheter versus surgical aortic valve implantation in patients with bicuspid aortic valve stenosis. EuroIntervention.

[106] Elbadawi A., Saad M., Elgendy I.Y. (2019). Temporal trends and outcomes of transcatheter versus surgical aortic valve replacement for bicuspid aortic valve stenosis. JACC Cardiovasc Interv.

[107] Mentias A., Sarrazin M.V., Desai M.Y. (2020). Transcatheter versus surgical aortic valve replacement in patients with bicuspid aortic valve stenosis. J Am Coll Cardiol.

[108] Kang J.J., Fialka N.M., El-Andari R. (2024). Surgical vs transcatheter aortic valve replacement in bicuspid aortic valve stenosis: a systematic review and meta-analysis. Trends Cardiovasc Med.

[109] Sá M.P., Jacquemyn X., Tasoudis P.T. (2022). Immediate and late outcomes of transcatheter aortic valve implantation versus surgical aortic valve replacement in bicuspid valves: meta-analysis of reconstructed time-to-event data. J Card Surg.

[110] Jørgensen T.H., Savontaus M., Willemen Y. (2025). Three-year-follow-up of the NOTION-2 trial: TAVR versus SAVR to treat younger low-risk patients with tricuspid or bicuspid aortic stenosis. Circulation.

[111] Toff W.D., Hildick-Smith D., Kovac J. (2022). Effect of transcatheter aortic valve implantation vs surgical aortic valve replacement on all-cause mortality in patients with aortic stenosis: a randomized clinical trial. Jama.

[112] Jad Malas S.A.P., Chen Q., Wen C. (2024). https://www.tctmd.com/slide/guidelines-versus-practice-statewide-survival-analysis-savr-versus-tavr-patients-aged-60.

[113] Varc Writing C., Genereux P., Piazza N. (2021). Valve academic research consortium 3: updated endpoint definitions for aortic valve clinical research. J Am Coll Cardiol.

[114] Jüni P., Antoniou S., Arbelo E. (2025). 2024 revision of the level of evidence grading system for ESC clinical practice guideline recommendations I: therapy and prevention. Eur Heart J.

[115] Van Mieghem N.M., van Gils L., Ahmad H. (2016). Filter-based cerebral embolic protection with transcatheter aortic valve implantation: the randomised MISTRAL-C trial. EuroIntervention.

[116] Haussig S., Mangner N., Dwyer M.G. (2016). Effect of a cerebral protection device on brain lesions following transcatheter aortic valve implantation in patients with severe aortic stenosis: the CLEAN-TAVI randomized clinical trial. JAMA.

[117] Kapadia S.R., Makkar R., Leon M. (2022). Cerebral embolic protection during transcatheter aortic-valve replacement. N Engl J Med.

[118] Kharbanda RK, Kennedy J, Jamal Z (2025). Investigators. Routine Cerebral Embolic Protection during Transcatheter Aortic-Valve Implantation. N Engl J Med.

[119] Lansky A.J., Schofer J., Tchetche D. (2015). A prospective randomized evaluation of the TriGuard™ HDH embolic DEFLECTion device during transcatheter aortic valve implantation: results from the DEFLECT III trial. Eur Heart J.

[120] Nazif T.M., Moses J., Sharma R. (2021). Randomized evaluation of TriGuard 3 cerebral embolic protection after transcatheter aortic valve replacement: reflect II. JACC Cardiovasc Interv.

[121] Fan J., Fang X., Liu C. (2020). Brain injury after transcatheter replacement of bicuspid versus tricuspid aortic valves. J Am Coll Cardiol.

[122] Investigators U.T.T., Toff W.D., Hildick-Smith D. (2022). Effect of transcatheter aortic valve implantation vs surgical aortic valve replacement on all-cause mortality in patients with aortic stenosis: a randomized clinical trial. JAMA.

[123] Husso A., Airaksinen J., Juvonen T. (2021). Transcatheter and surgical aortic valve replacement in patients with bicuspid aortic valve. Clin Res Cardiol.

[124] Geißler J., Isham E., Hickey G., Ballard C., Corbett A., Lubbert C. (2022). Patient involvement in clinical trials. Commun Med (Lond).

[125] The Lancet Healthy L. (2024). Increasing patient and public involvement in clinical research. Lancet Healthy Longev.

