# Supplementary material for: Transcatheter Treatment of Bicuspid Aortic Valve Stenosis: From Observational Studies to Randomized Clinical Trials
Source: Struct Heart. 2025 Nov 10;9(12):100754. doi: 10.1016/j.shj.2025.100754 (PMC12718148; doi:10.1016/j.shj.2025.100754)
Supplement: Supplementary Tables [file mmc1.docx]

**Supplementary Table 1:** Surgical Aortic Valve Replacement (SAVR) in Bicuspid Aortic Valve (BAV) vs. Tricuspid Aortic Valve (TAV)

| **Study** | **N** | **Age (mean±SD)** | **Female (n (%))** | **Prostheses Type** | **Outcomes** | **Follow-up** | | | | | | | | |
| --- | --- | --- | --- | --- | --- | --- | --- | --- | --- | --- | --- | --- | --- | --- |
|  |  |  |  |  |  | **In-hospital** | **30-day** | **1-year** | **2-years** | **3-years** | **4-years** | **5-years** | **9-years** | **10-years** |
| **Single-Center Study**  Huntley (2018)  PS-matching study [2010-2012] * (1) | 198 vs. 198 | 68 ± 7 vs. 68± 5 | 64 (32%) | Mechanical: 19% (38/198)  Bioprosthesis: 81% (160/198) | **All-cause death** |  |  |  |  |  |  | 21% vs. 39% (P=0.02) |  |  |
| **Multi-Center Study**  Holmgren 2020  [2005-2016] (2) | 1142 vs. 1740 | 63 (56-71) vs. 75 (68–79) | 331 (29%) | Mechanical: 36.5% (417/1142)  Bioprosthesis: 63.5% (725/1142) | **All-cause death** |  |  |  |  |  |  | Relative mortality ratio 0.62 (95% CI, 0.50-0.77, P<0.001) in favor of bicuspid aortic valve (F/U: 4.6 years) |  |  |
| **Single-Center Study**  Haunschild (2020)  PS-matching study [2000-2019] ¥ (3) | 992 vs. 992 | 61.8 ± 11 vs. 61.9 ± 10 | 337 (34.0%) vs. 336 (33.9%) | Mechanical: 22.0% vs. 23.0%  Bioprosthesis:  78.0% vs. 77.0% | **All-cause death** | 1.0% vs. 1.0% |  | 3.0% [95% CI,2.0%-4.0%] vs. 3.0% [95% CI,2.0%-4.0%] |  |  |  | 6.0% [95% CI,4.0%-8.0%] vs. 10.0% [95% CI,8%-13%] | 11.0% [95% CI,7.0%-15.0%] vs. 18.0% [95% CI,14.0%-22.0%] |  |
|  |  |  |  |  | **Re-operation overall** | 11.0% vs. 10.0% (P = 0.7) |  | 4.0% [95% CI,2.0%-5.0%] vs. 2.0% [95% CI,1.0%-3.0%] |  |  |  | 6.0% [95% CI,4.0%-8.0%] vs. 7.0% [95% CI,5.0%-9.0%] | 15.0% [95% CI,12.0%-17.0%] vs. 14.0% [95% CI,10.0%-18.0%] |  |
| **Single-Center Study**  Coti (2022)  [2010-2020]* (4) | 107 vs. 690 | 67.8 ± 8.4 vs. 74.4 ± 7.0 | 49 (45.8%) | Rapid-deployment valve | **All-cause death** |  | 0.6% |  |  |  |  | 8% [95% CI,3%-9%] vs. ~20% |  | 12% [95% CI, 5%-27%] vs. ~50% (log-rank test P = 0.002) |
|  |  |  |  |  | **Aortic valve reintervention** |  |  | 1.2% [95% CI, 0.1%-5.7%] vs. 2.4% [95% CI, 1.4%-3.9%] |  |  |  | 4.9% [95% CI, 1.2%-12.6%] vs. 3.3% [95% CI, 1.9%-5.1%] |  | 12.1% [95% CI, 2.2%-31.2%] vs. 5.2% [95% CI, 2.7%-8.9%] |
| **Single-Center Study**  Çelik (2022)  PS-matching study [1987-2016] † (5) | 593 vs. 593 | 61.0 ± 11.7 vs. 61.6 ± 12.5 | 198 (33.4%) vs. 193 (32.5%) | Mechanical: 63.4% (376/593)  Bioprosthesis: 26.6% (217/593) | **All-cause death** |  |  |  |  |  |  | 6.9% vs 11.3% |  | 14.9% vs 30.1% |
| **Single-Center Study**  Wedin (2022)  [2014-2021] (6) | 152 vs. 119 | 65.0 ± 9.1 vs. 71±6.7 | 52 (34%) | Not specified | **All-cause death** |  |  |  |  |  |  |  |  | 6 vs. 11 deaths during follow-up (1269 vs. 1441 days) |
|  |  |  |  |  | **Re-hospitalization** |  |  |  |  |  |  |  |  | 24 vs. 7 hospitalizations during follow-up (1269 vs. 1441 days) |
| **STS Adult Cardiac Database**  Hirji (2023) [2011-2018] (7) | 9131 vs. 56556 | 70 (67-74) vs.  75 (70-80) | 3785 (41.5%) vs. 25886 (45.8%) | Mechanical: 2.6% (241/9131)  Bioprosthesis: 91.2% (8327/9131) | **All-cause death** |  |  | 3.2% vs. 6.5% | 4.8% vs. 9.9% | 6.6% vs. 13.9% | 9.3% vs. 19.0% | 12.5% vs. 25.0% (p<0.0001) |  |  |
|  |  |  |  |  | **Re-hospitalization** |  |  | 8.8% vs. 8.9% | 11.7% vs. 12.2% | 13.6% vs. 15.6% | 16.3% vs. 18.9% | 18.9% vs. 22.5% (HR 0.88 (0.83-0.95)) |  |  |
| **Single-Center Study**  Im (2023)  [2016-2022]*(8) | 147 vs. 105 | 53 ± 36.1 vs. 60 ± 57.1 | 53 (36.1%) | Edwards Intuity:100% | **All-cause death** |  | 2.0%^§^ |  |  |  |  |  |  |  |
| **Single-Center Study**  Makkinejad (2025)  PS-matching study  [2000-2022]¥ (9) | 182 vs. 182 | 66 (60-72) vs. 70 (64-77) |  | Bioprosthesis only | **All-cause death** |  |  |  |  |  |  |  |  | 22% [95% CI, 15%-31%] vs. 38% [95% CI, 30%-48%] (P=0.004) |
| **Single-Center Study**  Makkinejad (2025)  PS-matching study  [2000-2022]*(9) | 350 vs. 350 | 67 (61-73) vs. 70 (63-75) | 132 (38%) | Bioprosthesis only | **All-cause death** |  |  |  |  |  |  |  |  | 33% [95% CI, 26%-41%] vs. 46% [95% CI, 39%-54%] (P=0.001) |
|  | * including concomitant surgery  † including CABG  ¥ excluding concomitant surgery  § operative mortality  CABG = coronary artery bypass grafting; F/U = follow-up; HR = hazard ratio; PS = propensity score; SAVR = surgical aortic valve replacement | | | | | | | | | | | | | |

**Supplementary Table 2:** Surgical Aortic Valve Replacement (SAVR) in Bicuspid Aortic Valve (BAV) and Concomitant Aortopathy

| **Study** | **N** | **Age (mean±SD)** | **Female (n (%))** | **Prostheses Type** | **Outcomes** | **Follow-up** | | | | | | | |
| --- | --- | --- | --- | --- | --- | --- | --- | --- | --- | --- | --- | --- | --- |
|  |  |  |  |  |  | **30-day** | **1-year** | **2-years** | **3-years** | **4-years** | **5-years** | **10-years** | **15-years** |
| **Single-Center Study**  Rinewalt (2014)  PS-matching study [2004-2011]  SAVR (Aorta <45mm) vs. SAVR/Aortic (Aorta 45-49mm) * (10) | 47 vs. 47 | Not specified | Not specified | Not specified | **All-cause death** |  | 0% vs. 4.3% | 2.1% vs. 4.3% | 2.1% vs. 4.3% | 6.0% vs. 4.3% | 6.0% vs. 4.3% |  |  |
| **Single-Center Study**  Rinewalt (2014)  PS-matching study [2004-2011]  SAVR (Aorta <45mm) vs. SAVR/Aortic (Aorta ≥50mm) *(10) | 62 vs. 62 | Not specified | Not specified | Not specified | **All-cause death** |  | 3.2% vs. 8.1% | 4.8% vs. 9.7% | 4.8% vs. 9.7% | 7.3% vs. 11.9% | 10.1% vs. 11.9% |  |  |
| **Single-Center Study**  Rinewalt (2014)  PS-matching study [2004-2011]  SAVR (Aorta 45-49mm) vs. SAVR/Aortic (Aorta ≥50mm) *(10) | 36 vs. 36 | Not specified | Not specified | Not specified | **All-cause death** |  | 2.8% vs. 5.6% | 2.8% vs. 5.6% | 2.8% vs. 5.6% | 2.8% vs. 9.8% | 2.8% vs. 9.8% |  |  |
| **Single-Center Study**  Svensson (2017) [1993-2003] Aortic valve surgery † vs. Aortic valve surgery/aortic repair (11) | 361 vs. 1459 | Not specified | 66 (18%) | Mechanical: 21% (68/322)  Bioprosthesis: 78% (250/322) | **All-cause death** | 1.1% (4) |  |  |  |  |  |  |  |
|  |  |  |  |  | **Stroke** | 1.7% (6) |  |  |  |  |  |  |  |
| **Single-Center Study**  Kaneko (2018) [2002-2014] §(12) | 618 vs. 683 | Not specified | 151 (24%) | Mechanical: 26% (143/550)  Bioprosthesis: 74% (407/550) | **All-cause death or reoperation** |  | 2.7% |  |  |  | 6.5% |  |  |
| **Single-Center Study**  Celik (2021)  [1987-2016]  SAVR Ψ and concomitant aortic surgery (13) | 48 | 58.7 ± 13.2 | 16 (33.3%) | Mechanical: 60% (29/48)  Bioprosthesis: 40% (19/48) | **All-cause death or reoperation** |  | 1% |  |  |  | 4% |  |  |
| **Single-Center Study**  Brown (2021)  [1992-2013]  SAVR and aortic replacement in BAV vs. TAV  (14) | 330 vs. 330 | 62 (52-71) | 97 (29%) | Freestyle Porcine Aortic root: 100% | **All-cause death** | 1.8% vs. 1.2% (p=0.52) |  |  |  |  |  | 72% [95% CI, 65%-77%], 59% [95% CI, 52%-65%] | 46% [95% CI, 38%-54%], 33% [95% CI, 26%-41%] |
|  |  |  |  |  | **Aortic valve reintervention** |  |  |  |  |  |  |  | 15% [95% CI, 10%-22%], 11% [95% CI, 6.4%-17%] |
| * Patients in the <45mm group mostly received a replacement of the aortic valve (96%), patients in the 45-49mm and ≥50mm group received root reconstruction with a valve conduit in 80% and 81% of the cases, respectively.  † including aortic valve repair  § including CABG and mitral valve surgery  Ψ including CABG  CABG = coronary artery bypass grafting; F/U = follow-up; PS = propensity score; SAVR = surgical aortic valve replacement | | | | | | | | | | | | | |

**Supplementary Table 3:** Transcatheter Aortic Valve Implantation **(**TAVI) in Bicuspid Aortic Valve (BAV) Stenosis

| **Study**  (year of publication) [recruitment] | **N** | **Sievers** type | **Female** | **Age (years)** mean±SD | **STS-PROM** (mean±SD or median (IQR)) | **Type of THV device** | **Outcomes** | **Follow-up** | | | | | | | |
| --- | --- | --- | --- | --- | --- | --- | --- | --- | --- | --- | --- | --- | --- | --- | --- |
| **Low risk patients** |  |  |  |  |  |  |  | **(Peri)-procedural** | **In-hospital (discharge)** | **30-day** | **1-year** | **2-year** | **3-year** | **5-year** | |
| **Multicenter international real-world registry** (14 countries)  Jia (2025)(15)  [2015-2022] | 894 | Type 0 22.8%,  Type 1 75.2%, Type 2 2.0% | 39.1% | 75.6 ± 8.1 | 3.9 ± 3.2 | SE THV 64.8%, BE THV 30.8%, mechanically‐  expandable THV4.4% | **Bioprosthetic valve failure** (composite endpoint of severe structural valve deterioration (SVD), valve‐related death, and aortic valve reintervention) |  |  |  |  |  |  | 6.1% | |
|  |  |  |  |  |  |  | **Valve-related death** |  |  |  |  |  |  | 0 | |
|  |  |  |  |  |  |  | **Aortic valve reintervention** |  |  |  |  |  |  | 2.4% | |
|  |  |  |  |  |  |  | **Hemodynamic & Echo outcomes** |  |  |  |  |  |  | ≥Moderate intraprosthetic AR: 1.7%  Severe PVL: 0.5%  Ψ Moderate‐to‐severe SVD: 8.1%  Ψ Severe SVD: 3.2% | |
| **Evolut Low Risk Bicuspid Study**  Zahr (2024**)**(16)  Forrest (2021)(17)  [2018-2019] | 150 | Type 0 9.3%,  Type 1 90.7%, Type 2 0% | 48.0% | 70.3 ± 5.5 | 1.4 ± 0.6 | Evolut R and PRO (100.0%) | **All-cause death** |  |  | 0.7% | 1.0% | 3.0% | 4.0% |  | |
|  |  |  |  |  |  |  | **Stroke** |  |  | 4.0% | 7.0% | 9.0% | 10.0% |  | |
|  |  |  |  |  |  |  | ***Re-hospitalization** |  |  | 4.0% | 5.0% | 8.0% | 8.0% |  | |
|  |  |  |  |  |  |  | **Aortic valve reintervention** |  |  | 0 | 1.0% | 2.0% | 2.0% |  | |
|  |  |  |  |  |  |  | **Functional outcomes** |  |  |  | 21.3 points+ in KCCQ | 18.9 points+ in KCCQ | 18.7 points+ in KCCQ |  | |
|  |  |  |  |  |  |  | **Hemodynamic & Echo outcomes** |  |  | Mean gradient (mmHg) >20: 1.4% | ≥ Moderate PVL:0.8%  Mean  Gradient (mmHg): 8.6±3.9 | ≥ Moderate PVL: 0  Mean  Gradient (mmHg): 8.7±3.7 | ≥ Moderate PVL: 0  Mean Gradient (mmHg): 9.1±5.8 |  | |
| **BIVOLUTX registry**  Tchétché (2023) [2018-2020](18) | 149 | Type 0 10.1%,  Type 1 86.6% (Type 1 L-R 72.5%),  Type 2 3.3% | 36.9% | 78.4±7.5 | 2.6 (1.7-4.2) | Evolut R and PRO (100.0%) | **All-cause death** | 0.7% |  | 4.0% | 11.0% |  |  |  | |
|  |  |  |  |  |  |  | **Stroke** |  |  | 4.7% (stroke or TIA) | 6.6% (stroke or TIA) |  |  |  | |
|  |  |  |  |  |  |  | ***Re-hospitalization** |  |  | 10.7% | 27.9% |  |  |  | |
|  |  |  |  |  |  |  | **Aortic valve reintervention** | 2.0%  received >1 THV due to malpositioning |  | 1.4% (new cardiac intervention) | 2.1% (new cardiac intervention) |  |  |  | |
|  |  |  |  |  |  |  | **Hemodynamic & Echo outcomes** | Mean gradient (mmHg): 4.0 (0-7) |  | ≥ Moderate AR: 2.8% | ≥ Moderate AR: 3.5%  Mean gradient (mmHg): 8.1 (6.2-11.1) |  |  |  | |
| **PARTNER 3 Bicuspid Registry**  Williams (2022)  [2016-2017](19) | 148 | Type 0 13.6%. Type 1 85.8%, Type 2 0.6% | 41.9% | 71.0 (68.0-75.0 | 1.4 (1.0-1.9) | Sapien 3 (100.0%) | **All-cause death** |  |  | 0 | 0.7% vs. 1.4%  (P = 0.58) |  |  |  | |
|  |  |  |  |  |  |  | **Stroke** |  |  | 1.4% | 2.0% vs. 2.1%  (P = 0.99) |  |  |  | |
|  |  |  |  |  |  |  | **Re-hospitalization** |  |  | 5.4% | 9.6% vs. 9.5% (P = 0.96) |  |  |  | |
|  |  |  |  |  |  |  | **Functional outcomes†** |  |  | 91.4† | 92.3)† |  |  |  | |
| **Low Risk TAVR trial**  Waksman (2020) [2016-2019](20) | 61 | Type 0 14.0%, Type 1 82.5%, Type 2 3.5% | 57.4% | 68.6 ± 7.4 | 1.5 ± 0.6 | Balloon-expandable (Sapien 3) 74.0%, Self-expanding (Evolut R or Evolut PRO) 26.0% | **All-cause death** |  | 0 | 0 |  |  |  |  | |
|  |  |  |  |  |  |  | **Stroke** |  | 1.6% | 1.6% |  |  |  |  | |
|  |  |  |  |  |  |  | **Hemodynamic & Echo outcomes** |  | ≥ Moderate PVL: 0  Mean gradient 15.1±5.2mmHg | ≥ Moderate PVL: 2.0%  Mean gradient 13.1±4.1 |  |  |  |  | |
| **Multicenter study**  Attinger‐Toller (2019) [2012-2017](21) | 79 | Type 0 6.0%, Type 1 81.0%, Type 2 5.0%, Undetermined 8.0% | 44.0% | 76 ± 9 | 3.8 (2.3–5.5) | Sapien 3 (100.0%) | **All-cause death** | 1.3% |  | 3.8% | 7.7% |  |  |  | |
|  |  |  |  |  |  |  | **Stroke** | 1.3% |  | 1.3% | 1.3% |  |  |  | |
|  |  |  |  |  |  |  | ***Re-hospitalization** |  |  | 0 | 2.6% |  |  |  | |
|  |  |  |  |  |  |  | **Aortic valve reintervention** | Surgical conversion: 1.3% |  | 0 | 0 |  |  |  | |
|  |  |  |  |  |  |  | **Functional outcomes** |  |  | 9.0% NYHA III (no patient NYHA IV) | 15-month:  7.0% NYHA III (no patient NYHA IV) |  |  |  | |
|  |  |  |  |  |  |  | **Hemodynamic & Echo outcomes** | Mean gradient>20mmHg: 2.8% |  | ≥ Moderate AR:0  Mean Gradient: 12.2 ± 4.3 | 15-month:  ≥ Moderate AR:0  Mean gradient: 14.5 ± 9.6 mmHg |  |  |  | |
| **Intermediate risk patients** | **N** | **Sievers** type | **Female** | **Age (years)** mean±SD | **STS-PROM** (mean±SD or median (IQR)) | **Type of THV device** | **Outcomes** | **(Peri)-procedural** | **In-hospital (discharge)** | **30-day** | **1-year** | **2-year** | **3-year** | | **4-year** |
| **Single-center study**  Boiago (2024**)** [before 2020]  (22) | 150 | Type 0 6.7%,  Type 1 92.6%, Type 2 0.7% | 32.7% | 81.4 ± 7.5 | 5.4 ± 6.4 | CoreValve (10.0%), Evolut R (26.0%), Evolut PRO (19.3%), Sapien 3 (44.77%) | **All-cause death** | 1.3% |  | 3.4% | 12.9% |  | 28.1% | |  |
|  |  |  |  |  |  |  | **Stroke** |  |  | 2.0% | 6.1% |  | 9.6% | |  |
|  |  |  |  |  |  |  | **Aortic valve reintervention** | Surgical conversion: 0.7% |  | 0 | 0 |  |  | |  |
|  |  |  |  |  |  |  | **Hemodynamic & Echo outcomes** | ≥ Moderate PVL (angio): 5.3% |  | ≥ Moderate PVL: 9.9%  Mean Gradient >20mmHg: 2.8% | ≥ Moderate PVL: 12.6%  Mean Gradient >20mmHg: 2.1% |  | ≥ Moderate PVL: 5.5%  Mean Gradient >20mmHg: 0 | |  |
| **STABILITY registry**  Fiorina (2023) [2011-2017](23) | 109 | Jilaihawi classification(24): Bicomissural non raphe type 7.3%, bicomissural raphe type 77.1%, tricomissural 15.6% | 50.0% | 78 ± 7.5 years | 5.1 ± 4.3 | Supra‐annular THVs (Evolut platform, Acurate neo) 53.2%, Intra-annular THVs (Sapien platform, Lotus, Portico) 46.8% | **All-cause death** | 0.9% |  |  |  |  |  | | 32.0% |
|  |  |  |  |  |  |  | **Stroke** | 3.7% |  |  |  |  |  | |  |
|  |  |  |  |  |  |  | **Aortic valve reintervention** | Implantation of a 2^nd^ THV: 3.7% |  |  |  |  |  | |  |
|  |  |  |  |  |  |  | **Hemodynamic & Echo outcomes** | >Moderate PVL: 13.1% |  | ≥ Moderate AR: 13.1%  Mean gradient (mmHg): 10.0 ± 5. | ≥ Moderate AR: 13.6%  Mean gradient (mmHg): 11.0 ± 6.0 | ≥ Moderate AR: 14.1%  Mean gradient (mmHg): 11.0 ± 5.5 | ≥ Moderate AR: 12.5%  Mean gradient (mmHg): 13.0 ± 6.0 | | ≥ Moderate AR: 6.8%  Mean gradient (mmHg): 13.0 ± 6.5 |
| **BEAT registry**  Mangieri (2020) [2013-2018](25) | 353 | Type 0 7.1%, Type 1 61.8%, Type 2 0.9% | 35.1% | 77.8 ± 8.3 | 4.4 ± 3.3 | Sapien 3 68.6% and Evolut R/PRO 31.4% | **All-cause death** | 1.1% |  | 4.3% |  |  |  | |  |
|  |  |  |  |  |  |  | **Stroke** |  |  | 1.6% |  |  |  | |  |
|  |  |  |  |  |  |  | ***Re-hospitalization** |  |  | 2.2% |  |  |  | |  |
|  |  |  |  |  |  |  | **Hemodynamic & Echo outcomes** | ≥ Moderate AR:  4.0% |  |  | ≥ Moderate AR:  6.2%  Mean gradient>20mmHg: 3.5% |  |  | |  |
| **Single-center study**  Lei (2019) [2012-2017](26) | 71 | Type 0 100% | 54.9% | 71.9 ± 5.8 | 7.0 ± 3.6 | CoreValve 22.5%, Venus-A 46.5%, VitaFlow 8.5%, Lotus 22.5% | **All-cause death** |  |  | 7.0% | 8.5% |  |  | |  |
|  |  |  |  |  |  |  | **Stroke** | 4.2% |  |  |  |  |  | |  |
|  |  |  |  |  |  |  | **Hemodynamic & Echo outcomes** | ≥ Moderate PVL: 0  Mean gradient: 15.6 ± 6.7 mmHg |  |  |  |  |  | |  |
| **Single-center study**  Yoon (2017) [2012-2016](27) | 108 | Type 0 5.6%, Type 1 94.4%, Type 2 0% | 28.7% | 74.4 ± 10.6 | 5.2 ± 3.4 | Sapien XT 31.5%, Sapien 3 68.5% | **All-cause death** |  |  | 0.9% | 6.9% |  |  | |  |
|  |  |  |  |  |  |  | **Stroke** |  |  | 4.6% |  |  |  | |  |
| **Multicenter study**  Perlman (2016) [2012-2015](28) | 51 | Type 0 11.8%, Type 1 74.7%, Type 2 1.9%, Functional 7.8%, Undetermined 3.8% | 52.9% | 76.2 ± 9.3 | 5.2 ± 3.7 | Sapien 3 (100.0%) | **All-cause death** |  |  | 3.9% |  |  |  | |  |
|  |  |  |  |  |  |  | **Stroke** |  |  | 1.9% |  |  |  | |  |
|  |  |  |  |  |  |  | **Aortic valve reintervention** | 0 surgical conversion |  |  |  |  |  | |  |
|  |  |  |  |  |  |  | **Hemodynamic & Echo outcomes** | Mean gradient >20mmHg: 1.9% |  | ≥ Moderate AR: 0  Mean gradient: 11.2 ± 4.7mmHg |  |  |  | |  |
| **Bicuspid TAVR registry**  Yoon (2016) [2005-2015](29) | 301 | Type 0 11.9%, Type 1 86.2%, Type 2 1.9%, undetermined 13.6% | 42.5% | 77.0 ± 9.2 | 4.7 ± 5.2 | Sapien XT 18.9%, Sapien 3 30.2%, CoreValve 37.2%, Lotus 3.7% | **All-cause death** | 1.3% |  | 4.3% | 14.4% | 17.9% |  | |  |
|  |  |  |  |  |  |  | **Stroke** |  |  | 2.3% |  |  |  | |  |
|  |  |  |  |  |  |  | **Aortic valve reintervention** | Surgical conversion: 2.9% |  |  |  |  |  | |  |
|  |  |  |  |  |  |  | **Hemodynamic & Echo outcomes** | ≥ Moderate PVL: 5.6%  Mean gradient ≥20mmHg: 4.7% |  |  |  |  |  | |  |
| **Multicenter study**  Jilaihawi (2016) [2005-2014](24) | 130 | **TAVI-directed classification**:  Tricommissural 23.5%, Bicommissural raphe-type 55.6%, Bicommissural non-raphe type 21.1% | 38.5% | 76.6 ± 10.4 | 4.7 (3.0-7.3) | Sapien 13.1%, Sapien XT 34.6%, Sapien 3 6.2%, Corevalve 46.2% | **All-cause death** | 1.5% |  | 3.8% |  |  |  | |  |
|  |  |  |  |  |  |  | **Stroke** |  |  | 3.2% |  |  |  | |  |
|  |  |  |  |  |  |  | **Aortic valve reintervention** | Surgical conversion: 3.1% |  |  |  |  |  | |  |
|  |  |  |  |  |  |  | **Hemodynamic & Echo outcomes** | ≥ Moderate PVL: 18.1%  Mean gradient (mmHg): 9.3 [7.0–13.0] |  |  |  |  |  | |  |
| **Multicenter study**  Mylotte (2014) [2005-2014](30) | 139 | Type 0 26.7%, Type 1 68.3%, Type 2 5.0% | 43.9% | 78.0 ± 8.9 | 4.9 ± 3.4 | Sapien 34.5%, CoreValve 65.5% | **All-cause death** | 3.6% |  | 5.0% | 17.5% |  |  | |  |
|  |  |  |  |  |  |  | **Stroke** |  |  |  | 2.2% |  |  | |  |
|  |  |  |  |  |  |  | **Aortic valve reintervention** | Surgical conversion: 2.2% |  |  |  |  |  | |  |
|  |  |  |  |  |  |  | **Hemodynamic & Echo outcomes** | ≥ Moderate AR: 28.4%  Mean gradient 11.4± 9.9 mmHg |  |  |  |  |  | |  |
| **High risk patients** | **N** | **Sievers** type | **Female** | **Age (years)** mean±SD | **STS-PROM** (mean±SD or median (IQR)) | **Type of THV device** | **Outcomes** | **(Peri)-procedural** | **In-hospital (discharge)** | **30-day** | **1-year** | **2-year** | **3-year** | | |
| **Multicenter study**  Kochman (2019) [2015-2016](31) | 24 | Type 0 8.0%, Type 1 75.0%, Type 2 0%, Functional 8%, Undetermined 8% | 50.0% | 75.3 ± 7.9 | 13.4 ± 10.4 (Logistic EuroScore) | Lotus (100.0%) | **All-cause death** |  |  | 4.0% |  | 12.5% |  | | |
|  |  |  |  |  |  |  | **Stroke** |  |  | 4.0% |  | 4.2% |  | | |
|  |  |  |  |  |  |  | **Hemodynamic & Echo outcomes** |  |  | ≥ Moderate PVL: 9.0%  Mean gradient: 14±4.3 mmHg |  | ≥ Moderate PVL: 0  Mean gradient: 15.8±10 mmHg |  | | |
| **Multicenter study**  Yousef (2015) [2005-2014](32) | 108 | Type 0 16.7%, Type 1 73.0%, Type 2 10.3% | 32.1% | 75.5 ± 14.4 | 17.2 ± 12.2 (Logistic EuroScore) | Sapien 56.5%, CoreValve 43.5% | **All-cause death** |  |  | 8.3% | 16.9% |  |  | | |
|  |  |  |  |  |  |  | **Stroke** |  |  | 2.8% |  |  |  | | |
|  |  |  |  |  |  |  | ***Re-hospitalization** |  |  | 2.3% | 13.3% |  |  | | |
|  |  |  |  |  |  |  | **Aortic valve reintervention** |  |  | 9.3% |  |  |  | | |
|  |  |  |  |  |  |  | **Functional outcomes** |  |  | 1.7% NYHA III or IV | 4.3% NYHA III or IV |  |  | | |
|  |  |  |  |  |  |  | **Hemodynamic & Echo outcomes** |  |  | ≥ Moderate AR: 30.8%  Mean gradient ≥20mmHg: 2.3% | ≥ Moderate PVL: 27.7%  Mean gradient ≥20mmHg: 6.7% |  |  | | |
| *Cardiac re-hospitalizations are reported and if not available, all-cause re-hospitalizations reported  † Reported as KCCQ overall summary score.  Ψ VARC-3 defitions  AR = Aortic regurgitation; BE = Balloon-Expandable; NYHA = New-York Heart Association; PVL = paravalvular leak; Self-Expanding; STS-PROM = Society of Thoracic Surgeons Predicted Risk of Mortality; SVD = structural valve deterioration; TAVI = transcatheter aortic valve implantation; THV = transcatheter heart valve; TIA = transient ischemic attack | | | | | | | | | | | | | | | |

**Supplementary Table 4:** Transcatheter Aortic Valve Implantation **(**TAVI) in Bicuspid Aortic Valve (BAV) vs. Tricuspid Aortic Valve (TAV)

| **Study**  (year of publication) [recruitment] | **N of BAV vs. TAV** | **Type of BAV** | **Female** | **Age** (mean±SD or median (IQR)) | **STS-PROM** (mean±SD or median (IQR)) | **Type of THV device** | **Outcomes***  **(bicuspid vs. tricuspid)** | **Follow-up** | | | | | |
| --- | --- | --- | --- | --- | --- | --- | --- | --- | --- | --- | --- | --- | --- |
| **Systemic Review & Meta-analysis** |  |  |  |  |  |  |  | **(Peri)-procedural** | **In-hospital (discharge)** | **30-day** | **1-year** | **~3.5-year** | |
| **Systemic Review & Meta-analysis**  Al-Asad (2023)  Subgroup analyses of the meta-analysis, **10 PS-matched** studies [2013-2023] Ψ (33) | 7’824 vs. 8’027 | Not specified | 36.8% vs. 36.2% | 72.0±3.2 vs. 72.1±3.5 | 3.5±1.7 vs. 3.6±1.8 | NA | **All-cause death** |  |  | OR 1.14, 95% CI 0.90 to 1.44,  P = 0.28, I² = 0% | 4.9% vs. 5.8% (OR 0.84, 95% CI 0.72 to 0.96, P = 0.01, I² = 0%) |  | |
|  |  |  |  |  |  |  | **Stroke** |  |  | 2.1% vs. 1.6%  (OR 1.38, 95% CI 1.09 to 1.75, P <0.05, I² = 0%) | OR 1.38, 95% CI 1.09 to 1.75, P <0.05 (I² = 0%) |  | |
| **Systemic Review & Meta-analysis**  Al-Asad (2023)  Subgroup analyses of the meta-analysis (**unmatched** population), **30 studies** [2013-2023] Ψ (33) | 8’286 vs. 8’384 | Not specified | 38.8% vs. 43.7% | 72.9±2.8 vs. 79.1±3.5 | 3.8±1.5 vs. 5.2±1.3 | Self-expanding | **All-cause death** |  |  | OR 1.18, 95% CI 0.77 to 1.81 | OR 0.86, 95% CI 0.63 to 1.17 |  | |
|  |  |  |  |  |  |  | **Stroke** |  |  | OR 1.55, 95% CI 1.02 to 2.36, P=0.04, I² =0% |  |  | |
|  |  |  |  |  |  | Balloon- expandable | **All-cause death** |  |  | OR 0.89, 95% CI 0.76 to 1.04 | OR 0.69, 95% CI 0.49 to 0.97 |  | |
|  |  |  |  |  |  |  | **Stroke** |  |  | OR 1.36, 95% CI 1.03 to 1.79, P = 0.03, I² = 0% |  |  | |
| **Systemic Review & Meta-analysis**  Montalto (2021)  6 studies reporting outcomes of **matched** populations [2013-2020] Ψ (34) | 3’434 vs. 3’637 | Not specified | 40.0% vs. 39.0% (P=0.671) | 76.7 (74.2-79.3) vs. 78.0 (71.7-81.4) (P=0.545) | 4.87 (4.61-5.13) vs. 5.00 (4.50-5.49) (P=0.650) | New generation: 52.0% vs. 54.0% (P=0.910)  Self-expanding:  55.0% vs. 50.0% (P=0.763) | **All-cause death** |  |  |  | RR: 0.91;  95% CI: 0.77-1.06; P = 0.23 |  | |
|  |  |  |  |  |  |  | **Stroke** | 2.43% (1.91-2.95) vs. 1.60 % (1.19-2.01) (P=0.015) |  |  |  |  | |
|  |  |  |  |  |  |  | **Surgical conversion** | 1.20% vs. 0.35% (P=0.018) |  |  |  |  | |
| **Systemic Review & Meta-analysis of reconstructed time-to-event data** (10 non-randomized and observational studies, 3 of them with matched populations)  Sá (2023) [2017-2022] Ψ (35) | 6’102 vs. 104’345 | Type 0: 42.7%  Type 1: 51.1%  Type 2: 3.4% | 33.3% to 47.7% vs. 35.8% to 47.2% (no statistical difference among the studies) | In 5  studies, BAV patients were younger than TAV (P<0.05) | In 5  studies, BAV patients were at lower risk than TAV (P<0.05) | BAV (average across 10 studies): 51.0% Balloon-expandable, 46.0% Self-expanding, 1,2% mechanically-expandable  TAV (only study 1 had values): 10.2% Balloon-expandable, 82.5% Self-expanding, 7.3% mechanically-expandable | **All-cause death** |  |  |  |  | Patients with BAV  had a significantly lower risk of mortality (HR 0.70, 95%CI 0.65–0.77, P<0.001), however, this result was  driven by populations in which the risk score was statistically significantly lower in the BAV group (HR  0.69, 95%CI 0.63–0.76, P<0.001) and by populations in which the BAV group was statistically significantly  younger (HR 0.72, 95%CI 0.64–0.81, P<0.001) | |
| **Propensity-Score matching studies** | **N of BAV vs. TAV** | **Type of BAV** | **Female** | **Age** (mean±SD or median (IQR)) | **STS-PROM** (mean±SD or median (IQR)) | **Type of THV device** | **Outcomes***  **(bicuspid vs. tricuspid)** |  |  |  |  |  |  |
| **Multicentre** |  |  |  |  |  |  |  | **(Peri)-procedural** | **In-hospital (discharge)** | **30-day** | **1-year** | **2-year** | **10-year** |
| **OCEAN-TAVI registry (Japan)**  Yamanaka (2025)  PS-matching study[2013-2019](36) | 497 vs. 497 | Not specified | 60.8% vs. 63.8% (P=0.33) | 84 (80-87) vs. 4 (80-87) (P=0.49) | 5.7 (3.6-8.7) vs. 6.0 (3.8-9.0) (P=0.55) | Self-expanding (CoreValve, Evolut R/PRO): 23.7% vs. 24.6% (no statistical significant difference)  Balloon-expandable (Sapien XT/3): 76.3% vs. 75.5% (no statistical significant difference) | **All-cause death** |  |  | 1.0% vs. 1.0% (P=1.0) |  |  |  |
|  |  |  |  |  |  |  | **Stroke** |  | 1.6% vs. 1.6% (P=1.0) |  |  |  |  |
|  |  |  |  |  |  |  | **Hemodynamic & Echo outcomes** |  | Moderate PPM, 11.7% vs 4.4%;  Severe PPM, 1.4% vs 1.0% (P = 0.0001)  ≥ Moderate PVL: 2.2% vs. 3.6% (P=0.27)  Mean gradient (mmHg): 11.0 (8.0-14.0) vs. 10.3 (7.85-13.1) (P=0.27) |  | Mean gradient (mmHg): 10 (7.6-13.0) vs. 10.0 (7.2-13.0) (P=0.73) | Mean gradient (mmHg): 10.0 (7.0-13.0) vs. 10.5 (7.23-13.2) (P=0.30) |  |
| **Low Risk Bicuspid trial and Evolut Low Risk Trial**  Deeb (2022)  PS-matching study[2016-2018](37) | 145 vs. 145 | Type 0 9.7%, Type 1 90.3% (no Type 0) | 46.9% vs. 49.0% (P=0.724) | 70.5 ± 5.5 vs. 71.2 ± 5.9 (P=0.321) | 1.4 ± 0.6 vs. 1.7 ± 0.7 (P=0.002) | Industry-sponsored (Medtronic): Evolut R or Evolut PRO | **All-cause death** | 0.7% vs. 0 (P=0.498) |  | 0.7% vs. 0 (P=0.317) | 0.7% vs. 2.1%  (P = 0.317) |  |  |
|  |  |  |  |  |  |  | **Stroke** |  |  | 4.1% vs. 2.1% (p=0.311) | 4.8% vs.2.8%  (P = 0.358) |  |  |
|  |  |  |  |  |  |  | **Re-hospitalization** |  |  | 2.1% vs. 0.7% (P=0.314) | 3.5% vs. 4.9% (P = 0.560) |  |  |
|  |  |  |  |  |  |  | **Aortic valve reintervention** |  |  | 0 vs. 0 | 0.7% vs. 0 (P = 0.317) |  |  |
|  |  |  |  |  |  |  | **Functional outcomes†** |  |  | 90.3 vs. 88.6  (P = 0.295) † | 92.8 vs. 91.0 (P = 0.212)† |  |  |
|  |  |  |  |  |  |  | **Hemodynamic & Echo outcomes** | ≥ Moderate PVL: 0 vs. 2.1% (P=0.121) |  | ≥ Moderate PVL: 0 vs. 4.7%  Mean gradient >20mmHg: 1.4% vs. 0 (P=0.498) | ≥ Moderate PVL: 0.8% vs 3.9%  Mean gradient >20mmHg: 1.6% vs. 0 (P=0.498) |  |  |
| **PARTNER 3 Bicuspid Registry**  Williams (2022)  PS-matching study [2016-2017](19) | 148 vs. 148 | In the non-matched population:  Type 0 13.6%. Type 1 85.8%, Type 2 0.6% | 41.9% vs. 39.9% | 71.0 (68.0-75.0) vs. 72.0 (68.0-  75.0) | 1.4 (1.0-1.9) vs. 1.5 (1.2-1.8) | Industry-sponsored (Edwards): Sapien 3 | **All-cause death** |  |  | 0 vs. 0 | 0.7% vs. 1.4%  (P = 0.58) |  |  |
|  |  |  |  |  |  |  | **Stroke** |  |  | 1.4% vs. 1.4% (P = 0.99) | 2.0% vs. 2.1%  (P = 0.99) |  |  |
|  |  |  |  |  |  |  | **Re-hospitalization** |  |  | 5.4% vs. 4.1% (P=0.58) | 9.6% vs. 9.5% (P = 0.96) |  |  |
|  |  |  |  |  |  |  | **Functional outcomes†** |  |  | 91.4 vs. 88.4  (P = 0.02)† | 92.3 vs. 91.6 (P = 0.50)† |  |  |
| **STS/ACC TVT registry**  Makkar (2021)  PS-matching study [2008-2016](38) | 3’168 vs. 3’168 | Not specified | 30.8% vs. 29.6% | 68.8 ± 8.7 vs. 68.7 ± 9.0 | 1.7 ± 0.6 vs. 1.7 ± 0.7 | Industry-sponsored (Edwards): Sapien 3 and Sapien 3 Ultra | **All-cause death** |  |  | 0.9% vs 0.8% (P=0.55) | 4.6% vs. 6.6%  (P = 0.06) |  |  |
|  |  |  |  |  |  |  | **Stroke** |  |  | 1.4% vs. 1.2% (P=0.55) | 2.0% vs 2.1% (P = 0.89) |  |  |
|  |  |  |  |  |  |  | **Functional outcomes†** |  |  | 84.3 vs. 84.2 (0.2 [-0.9, 1.2])† | 84.3 vs. 85.4 (-1.0 [-2.9, 0.8])† |  |  |
|  |  |  |  |  |  |  | **Hemodynamic & Echo outcomes** |  |  | ≥ Moderate PVL: 1.8% vs. 1.1% (P=0.07)  Mean gradient (mmHg): 12.7 ±5.4 vs. 12.9 ±5.6 (P=0.43) | ≥ Moderate PVL: 3.4% vs. 2.1% (P=0.33)  Mean gradient (mmHg): 13.2 ±6.0 vs. 13.5 ±6.1 (P=0.33) |  |  |
| **STS/ACC TVT registry**  Forrest (2020)  PS-matching study [2015-2018](39) | 929 vs. 929 | Not specified | 44.9% vs. 45.3% (P=0.85) | 73.0 ± 10.3 vs. 72.6 ± 10.8 (P=0.43) | 5.3 ± 4.2 vs. 5.2±4.1 (P=0.63) | Evolut R and Evolut PRO | **All-cause death** |  | 1.7% vs 0.8% (P=0.06) | 2.6% vs. 1.7% (P=0.18) | 10.4% vs. 12.4%  (P = 0.63) |  |  |
|  |  |  |  |  |  |  | **Stroke** |  | 2.9% vs. 2.6% (P=0.67) | 3.4% vs. 2.7% (P=0.41) | 3.9% vs. 4.4%  (P = 0.93) |  |  |
|  |  |  |  |  |  |  | **Re-hospitalization** |  |  | 1.1% vs. 0.7% (P=0.31) | 3.8% vs. 3.1% (P=0.40) |  |  |
|  |  |  |  |  |  |  | **Aortic valve reintervention** |  | Surgical conversion: 0.6% vs 0.2% (P=0.29) | 0.8% vs. 0.1% (P=0.03) | 1.7% vs. 0.3% (P=0.01) |  |  |
|  |  |  |  |  |  |  | **Functional outcomes†** |  |  |  | 78.9 vs. 78.6 (P = 0.87)† |  |  |
|  |  |  |  |  |  |  | **Hemodynamic & Echo outcomes** |  | ≥Moderate AR: 5.6% vs. 2.1% (P<0.001)  Mean gradient (mmHg): 9.7±5.2 vs. 9.0±5.0 (P=0.002) |  | ≥Moderate AR: 4.7% vs. 3.9% (P=0.60)  Mean gradient (mmHg): 9.4±5.2 vs. 8.9±5.1 (P=0.22) |  |  |
| **STS/ACC TVT registry**  Makkar (2019)  PS-matching study[2015-2018](40) | 2’691 vs. 2’691 | Not specified | 39.7% vs. 38.5% (ASD =0.025) | 74 (66-81) vs. 74 (66-81) (ASD =0.02) | 4.9 ± 4.0 vs. 5.1 ± 4.2 (ASD=0.047) | Industry-sponsored (Edwards): Sapien 3 | **All-cause death** |  | 1.7% vs. 1.6% (P=0.75) | 2.6% vs. 2.5% (P=0.82) |  |  |  |
|  |  |  |  |  |  |  | **Stroke** |  | 2.1% vs. 1.2% (P=0.01) | 2.5% vs. 1.6% (P=0.02) |  |  |  |
|  |  |  |  |  |  |  | **Re-hospitalization** |  |  | 0.6% vs. 0.7% (P=0.62) |  |  |  |
|  |  |  |  |  |  |  | **Surgical conversion** | 0.9% vs. 0.4% (P=0.03)  (annulus rupture: 0.3% vs. 0, P=0.02) |  |  |  |  |  |
|  |  |  |  |  |  |  | **Aortic valve reintervention** |  |  | 0.4% vs. 0.4% (P=0.99) |  |  |  |
|  |  |  |  |  |  |  | **Functional outcomes†** |  |  | 83.3 vs. 83.3 (P=0.44) † |  |  |  |
|  |  |  |  |  |  |  | **Hemodynamic & Echo outcomes** |  | ≥Moderate PVL: 1.5% vs 0.8% (P=0.04)  Mean gradient (mmHg): 11.6±5.7  vs 11.8±5.3 (P=0.15) | ≥Moderate PVL: 2.0% vs 2.4% (P=0.53)  Mean gradient (mmHg): 12.2±5.3  vs 12.3±5.4 (P=0.69) |  |  |  |
| **NIS database**  Elbadawi (2019)  PS-matching study [2012-2016](41) | 1’035 vs. 1’035 | Not specified | 39.1% vs. 38.6% | 68.4 ± 11.9 vs. 68.8 ± 12.2 | Not specified | Not specified | **All-cause death** |  | 2.9% vs. 3.4%  (P = 0.76) |  |  |  |  |
|  |  |  |  |  |  |  | **Stroke** |  | 1.9% vs. 1.9% (P>0.99) |  |  |  |  |
| **NIS database**  Nagaraja (2019)  PS-matching study [2011-2014](42) | 359 vs. 359 | Not specified | 65.2% vs. 62.4% (P=0.75) | 68.0 ± 13.4 vs. 68.2 ± 12.7 (P=0.95 | Not specified | Not specified | **All-cause death** |  | 5.6% vs. 1.4% |  |  |  |  |
|  |  |  |  |  |  |  | **Stroke** |  | 2.8% vs. 5.6% |  |  |  |  |
|  |  |  |  |  |  |  | **Surgical conversion** |  | 0 vs. 0 |  |  |  |  |
| **European ACURATE *neo* registry**  Mangieri (2018)  PS-matching study [2012-2017](43) | 54 vs. 54 | Type 0 3.7%, Type 1 90.7%, Type 2 1.8%, Undetermined 1.8% | 61.1% vs. 42.5% (P=0.154) | 80 ± 5.3 vs. 81.1±5.5 (P=0.221) | 4.7 ± 2.7 vs. 4.7 ± 2.8 (P=0.735) | ACURATE neo | **All-cause death** |  |  | 3.7% vs. 8.7%  (P = 0.295) |  |  |  |
|  |  |  |  |  |  |  | **Stroke** |  |  | 7.4% vs. 3.7%  (P = 0.401) |  |  |  |
|  |  |  |  |  |  |  | **Re-hospitalization** |  |  | 5.5% vs. 0% (P = 0.196) |  |  |  |
|  |  |  |  |  |  |  | **Hemodynamic & Echo outcomes** |  |  | ≥Moderate PVL: 3.1% vs. 5.5% (P=0.734)  Mean gradient (mmHg): 9.8 ± 4.2 vs. 9.9 ± 4.5 (P=0.944) |  |  |  |
| **Bicuspid TAVR registry** (Europe, North America, and  Asia-Pacific)  Yoon (2017)  PS-matching study [2013-2016](44) | 546 vs. 546 | Type 0 12.8%, Type 1 85.6%, Type 2 1.7%, Undetermined 12.5% | 37.2% vs. 39.4% (P=0.48) | 77.2 ± 8.2 vs. 77.2 ± 8.8 (P=0.91) | 4.6 ± 4.6 vs. 4.3 ± 3.0 (P=0.29) | “Early” generation  Devices (Sapien XT and CoreValve):  58.6% vs. 58.8% (P>0.99)  “New” generation devices (Sapien 3, Lotus, Evolut R):  41.4% vs. 41.2% (P>0.99) | **All-cause death** | 1.3% vs. 1.1% (P>0.99) |  | 3.7% vs. 3.3% (P=0.87) | “Early” generation devices: 14.5% vs. 13.7%; log-rank  p = 0.80;  “New” generation devices: 4.5% vs. 7.4%;  log-rank p = 0.64 | 17.2% vs. 19.4% (P = 0.28) |  |
|  |  |  |  |  |  |  | **Stroke** |  |  | 2.9% vs. 1.8% (P=0.33) |  |  |  |
|  |  |  |  |  |  |  | **Surgical conversion** | 2.0% vs. 0.2% (P=0.006) |  |  |  |  |  |
|  |  |  |  |  |  |  | **Hemodynamic & Echo outcomes** | ≥Moderate PVL: 10.4% vs. 6.8% (P=0.04)  Mean gradient (mmHg): 10.8 ± 6.7 vs. 10.2 ± 4.4 (P=0.18) |  |  |  |  |  |
| **Five centers in Poland**  Gasecka (2022)  1:3 PS-matching study [2009-2017](45) | 130 vs. 390 | Not specified | 40.0% vs 49.0% (P=0.068) | 79 (74–82) vs. 80 (76–84) (P=0.136) | EuroScore II:  3.6 (2.6–5.1) vs. 3.8 (2.8–6.5) (P=0.171) | SE THV 74.0% vs. 66.0% (P=0.115)  New generation: 44.0% vs. 30.0% (P<0.001) | **All-cause death** |  | 2.3% vs. 2.1% (P=0.863) |  |  |  | 22.5% vs. 23.7% (P=0.633) |
|  |  |  |  |  |  |  | **Stroke** | 5.0% vs. 2.0% (P=0.079) |  |  |  |  |  |
|  |  |  |  |  |  |  | **2^nd^ valve implantation** | 1.5% vs. 1.0% (P=0.635) |  |  |  |  |  |
|  |  |  |  |  |  |  | **Hemodynamic & Echo outcomes** |  | ≥ Moderate PVL: 2.0% vs. 2.0% (P=0.846)  Mean gradient (mmHg): 9 (7–13) vs. 10 (7–14) (P=0.165) |  |  |  |  |
| **Single-centre** | **N of BAV vs. TAV** | **Type of BAV** | **Female** | **Age** (mean±SD or median (IQR)) | **STS-PROM** (mean±SD or median (IQR)) | **Type of THV device** | **Outcomes***  **(bicuspid vs. tricuspid)** | **(Peri)-procedural** | **In-hospital (discharge)** | **30-day** | **1-year** | **2-year** | |
| **Single-centre registry**  He (2023)  Propensity tri-matched 1:1:1 study (Type 0 vs. Type 1 vs. Tricuspid) [2012-2022](46) | 121 Type 0 vs. 121 Type 1 vs. 121 Tricuspid | Type 0 50.0%, Type 1 50.0% | 37.2% vs. 37.2% vs. 39.7% (P=0.935) | 73.0±6.0 vs. 72.5±8.0 vs. 72.2±7.7 (P=0.105) | 5.2±3.9 vs. 5.0±4.1 vs. 4.7±3.1 (P=0.075) | First generation self-expanding 94.8%, Balloon-expandable 5.2% | **All-cause death** |  | 3.3% vs. 1.7% vs. 1.7% (P=0.739) | 4.2% vs. 1.7% vs. 1.7% (P=0.522) | 10.0% vs. 2.3% vs. 6.2%, (P=0.099) |  | |
|  |  |  |  |  |  |  | **Stroke** |  | 0.8% vs. 0.8% vs. 0 (P=0.776) | 1.0% vs.0.9% vs 0 (P=0.765) | 1.4% vs. 1.6% vs. 1.3% (P=NS) |  | |
|  |  |  |  |  |  |  | **2^nd^ valve implantation** | 15.8% vs. 11.7% vs. 9.1% (P=0.291) |  |  |  |  | |
|  |  |  |  |  |  |  | **Hemodynamic & Echo outcomes** |  | ≥ Moderate PVL: 6.7% vs. 6.6% vs. 3.3% (P=0.460)  Mean gradient (mmHg): 21.6 vs. 7.5 vs. 14.5 (P=0.008 | ≥ Moderate PVL: 13.2% vs. 14.5% vs. 1.5% (P=0.012)  Mean gradient (mmHg): 15.2 vs. 14.1 vs. 13.0 (P=0.120) | ≥ Moderate PVL: 15.6% vs. 6.2% vs. 6.1% (P=0.366)  Mean gradient: 5.7 vs. 6.7 vs. 5.9 (P=0.417 |  | |
| **Single-centre study**  Michel (2021) PS-matching (1:2) study [2014-2019](47) | 78 vs. 156 | Type 0 9.0%,  Type 1 91.0% | 38.5% vs. 44.8%  (P=0.34) ¥ | 77 (71-81) vs. 81 (78-85) (P<0.001) ¥ | EuroScore II:  2.96 [1.98, 4.43] vs. 4.51 [2.53, 7.87] (P<0.001) ¥ | Sapien 3 | **All-cause death** |  |  | 0 vs. 1.9% (P=0.20) | 1.3% vs. 7.3% (P=0.06) |  | |
|  |  |  |  |  |  |  | **Stroke** |  |  | 3.8% vs. 2.6% (P=0.60) | 3.8% vs. 4.7% (P=0.80) |  | |
|  |  |  |  |  |  |  | **Re-hospitalization** |  |  | 0 vs. 1.3% (P=0.30) | 5.3% vs. 4.0% (P=0.70) |  | |
|  |  |  |  |  |  |  | **Aortic valve reintervention** |  |  | 0 vs. 0.7% (P=0.50) | 0 vs. 2.7% (P=0.10) |  | |
| **Single-centre study**  De Biase (2018)  PS-matching (1:2) study [2015-2017](48) | 83 vs. 166 | Type 0 7.0%, Type 1 93.0% (no Type 2) | 31.0% vs. 34.0% (P=0.57) | 81.4 ± 7.6 vs. 82.9 ± 5.7 (P=0.07) | 5.1 ± 3.3 vs. 5.1 ± 2.9 (P=0.99) | Evolut R: 19.0% vs. 59.0%  Sapien 3 60.0% vs. 37.0%  Lotus: 4.0% vs. 2.0%  Portico 0 vs 0.6% | **All-cause death** |  | 3.0% vs. 2.0% (P=0.68) | 5.0% vs. 3.0%  (P = 0.47) |  |  | |
|  |  |  |  |  |  |  | **Stroke** |  | 0.5% vs. 1.0% (P = 1.0) | 0 vs. 0.6%  (P = 1.0) |  |  | |
|  |  |  |  |  |  |  | **Hemodynamic & Echo outcomes** |  | ≥Moderate PVL: 3.0% vs. 2.0% (P=0.48)  Mean gradient (mmHg): 9.8 ± 4.5 vs. 10.0 ± 4.0 (P=0.1) | ≥Moderate PVL: 5.0% vs. 3.0% (P=0.47)  Mean gradient (mmHg): 9.7 ± 4.8 vs. 10.4 ± 5.6 (P=0.44) |  |  | |
| **STS score–adjusted mortality** ¥  **Duke Aortic Valve Disease database**  Pineda (2020) [2011-2016](49) | 50 vs. 517 | Type 0 14.0%, Type 1 86.0% (no Type 2) | 36.0% vs. 44.1% (P=0.200) | 70 (64-74) vs. 81 (75-86) (P<0.01) | 4.6 (3.0-7.7) vs. 6.7 (4.4-9.9) (P=0.001) | CoreValve, Evolut R; Sapien, Sapien XT, or  Sapien S3 | **All-cause death** |  |  | 8.0% vs. 1.9% (P<0.001) ¥ | 15.0% vs. 11.0% (P=0.557) ¥ | 15.6% vs. 17.3%  (P = 0.110) ¥ | |
|  |  |  |  |  |  |  | **Stroke** |  | 2.0% vs. 1.5% (P=0.567) |  |  |  | |
|  |  |  |  |  |  |  | **Unplanned cardiac surgery** |  | 0 vs. 1.0% (P=0.629) |  |  |  | |
|  |  |  |  |  |  |  | **Hemodynamic & Echo outcomes** |  | ≥Moderate PVL: 4.0% vs 3.7% (P=0.568) |  |  |  | |
| **Non-adjusted studies** | **N of BAV vs. TAV** | **Type of BAV** | **Female** | **Age** (mean±SD or median (IQR)) | **STS-PROM** (mean±SD or median (IQR)) | **Type of THV device** | **Outcomes***  **(bicuspid vs. tricuspid)** |  |  |  |  |  | |
| **Multi-center** |  |  |  |  |  |  |  | **(Peri)-procedural** | **In-hospital (discharge)** | **30-day** | **1-year** | **2-year** | |
| **STS/ACC TVT registry**  Halim (2020) [2011-2018](50) | 5’412 vs. 165’547 | NA | 40.9% vs. 47.2% (P<0.001) | 74.0 (65.0–81.0) vs. 82.0 (76.0–87.0) (P <0.001) | 3.8 (2.3–6.1) vs. 5.6 (3.6–8.8) (P<0.001) | Sapien XT/3, CoreValve/Evolut | **All-cause death** |  | 2.0% vs. 2.2% (P=0.484) |  | HR 0.88 [95% CI, 0.78–0.99] |  | |
|  |  |  |  |  |  |  | **Stroke** |  | 2.2% vs. 1.9% (P=0.151) |  | HR 1.14 [95% CI, 0.94–1.39] |  | |
|  |  |  |  |  |  |  | **Surgical conversion** | 0.7% vs 0.6% |  |  |  |  | |
|  |  |  |  |  |  |  | **Hemodynamic & Echo outcomes** | ≥ Moderate AR: 4.7% vs. 3.5% (P=0.437) |  |  |  |  | |
| **BAVARD European Registry**  Tchétché (2019) [2017-2017](51) | 101 vs. 88 | Type 0 12.9%, Type 1 86.1%, Type 2 1.0% | 35.0% vs. 54.0% (P<0.001) | 78.2 ± 10.1 vs. 83.1±5.7 (P<0.001) | 11.3 ± 8.5 vs. 7.6±4.4 (P<0.001) | Sapien 3, Evolut R, Lotus | **All-cause death** |  |  | 0 vs. 3.4%  (P = 0.61) |  |  | |
|  |  |  |  |  |  |  | **Stroke** |  |  | 2.0% vs. 0%  (P = 0.5) |  |  | |
|  |  |  |  |  |  |  | **Hemodynamic & Echo outcomes** |  |  | ≥Moderate AR: 20.8% vs. 12.5% (P=0.11)  Mean gradient (mmHg): 10.7±4.9 vs. 9.4±4.9 (P=0.15) |  |  | |
| **Multicenter US study**  Sannino (2017) [2012-2016](52) | 88 vs. 735 | Type 0 13.6%, Type 1 85.2%, Type 2 1.1% | 39.8% vs. 47.1% (P=0.194) | 80.2 ± 8.4 | 7.4 ± 3.9% | Ballon-expandable (“early” and “new” generation): 52.3% vs. 59.8%  Self-expanding (“early” and “new” generation): 40.2% vs. 47.7% | **All-cause death** | 1.1% vs. 0.8% (P=0.757) |  | 3.4% vs. 3.1% (P=0.887) | 8.5% vs. 10.5%  (P = 0.579) |  | |
|  |  |  |  |  |  |  | **Stroke** | 2.3% vs. 3.7% (P=0.499) |  |  |  |  | |
|  |  |  |  |  |  |  | **Hemodynamic & Echo outcomes** | ≥Moderate PVL: 5.3% vs. 5.0% (P=0.903)  Mean gradient (mmHg): 8.0 ±4.1 vs. 8.5 ± 4.2 (P=0.268) |  |  | ≥Moderate PVL: 4.3% vs. 4.7% |  | |
| **Single-center** | **N of BAV vs. TAV** | **Type of BAV** | **Female** | **Age** (mean±SD or median (IQR)) | **STS-PROM** (mean±SD or median (IQR)) | **Type of THV device** | **Outcomes***  **(bicuspid vs. tricuspid)** | **(Peri)-procedural** | **In-hospital (discharge)** | **30-day** | **1-year** | **2-year** | **3-year** |
| **Single-center study**  Jin (2022)  **Type 0 vs. Type 1 vs. Tricuspid**  [2017-2019](53) | 86 vs. 109 vs. 149 | Type 0 44.1%, Type 1 55.9% | 52.3% vs. 49.5% vs. 48.4% (P=0.839) | 72.9±7.1 vs. 76.3±7.2 vs. 77.1±9.3 (P=0.001) | 4.2±1.8 vs. 4.5±1.6 vs. 5.1±2.2 (P< 0.001) | SE THVs (Venus-A, Vita-flow) | **All-cause death** |  | 2.3% vs. 0.9% vs. 2.0% (P=0.77) | 2.3% vs. 0.9% vs. 2.7% (P=0.626) | 3.5% vs. 3.7% vs. 5.4% (P=0.794) | 3.5% vs. 5.5% vs. 6.0% (P=0.782) |  |
|  |  |  |  |  |  |  | **Stroke** |  | 3.5% vs. 4.6% vs. 1.3% (P=0.273) | 4.7% vs. 5.6% vs. 2.0% (P=0.278) | 4.7% vs. 7.3% vs. 3.4% (P=0.315) | 4.7% vs. 7.3% vs. 4.0% (P=0.499) |  |
|  |  |  |  |  |  |  | **Re-hospitalization** |  |  | 2.3% vs. 0 vs. 0 (P=0.062) | 4.7% vs. 14.7% vs. 6.0% (P=0.016) | 12.8% vs. 20.2% vs. 8.7% (P=0.020) |  |
|  |  |  |  |  |  |  | **Aortic valve reintervention** | Implantation of a 2^nd^ valve:  6.7% vs 8.3% vs. 5.4% (P=0.651) |  | 0 vs. 0 vs. 0 | 0 vs. 0 vs. 0.7% (P≥0.999) | 0 vs. 0.9% vs. 0.7% (P≥0.999) |  |
|  |  |  |  |  |  |  | **Hemodynamic & Echo outcomes** | ≥Moderate PVL: 10.5% vs. 16.5% vs. 6.7% (P=0.043)  Mean gradient: 12.8±6.1 vs. 12.2±6.1 vs. 10.3±4.8 (P=0.005) |  |  |  |  |  |
| **Single-center study**  Zhou (2022)  [2013-2018](54) | 109 vs. 137 | Type 0 61.5%, Type 1 36.7%, Type 2 1.8% | 43.1% vs. 35.8% (P=0.240) | 75 (71-80) vs. 77 (73-81) (P=0.041) | 5.1 (3.7-8.6) vs. 6.0 (4.3-10.7) (P=0.026) | SE THVs: 83.5% vs. 82.5%,  BE THVs: 4.6% vs. 10.2%,  Mechanically expandable THVs: 11.9% vs. 7.3% (P=0.143) | **All-cause death** | Cardiopulmonary resuscitation: 4.6% vs. 2.2% (P=0.472) |  |  | 6.4% vs. 10.9% (P=0.216) | 10.1% vs. 16.1% (P=0.173) | 12.8% vs. 20.4% (P=0.116) |
|  |  |  |  |  |  |  | **Stroke** | 0.9% vs. 0.7% (P=1.000) |  |  | 4.6% vs. 2.2% (P=0.472) | 4.6% vs. 4.4% (P=1.000) | 7.3% vs. 4.4% (P=0.320) |
|  |  |  |  |  |  |  | **Aortic valve reintervention** |  |  |  | 0 vs. 1.5% (P=0.504) | 0 vs. 1.5% (P=0.504) | 0 vs. 1.5% (P=0.504) |
|  |  |  |  |  |  |  | **Functional outcomes** |  |  |  | NYHA III/IV: 27.5% vs. 45.9% (P=0.005) | 17.5% vs. 18.4% (P=0.866) | 13.0% vs. 15.9% (P=0.571) |
|  |  |  |  |  |  |  | **Hemodynamic & Echo outcomes** | ≥Moderate PVL: 6.5% vs. 11.9% (P=0.162),  Mean gradient (mmHg): 12.9±5.3 vs. 12.4±5.0 (P=0.423) |  |  | ≥Moderate PVL: 8.3% vs. 8.7% (P=0.925)  Mean gradient (mmHg): 11.4±5.8 vs. 10.4±4.4 (P=0.177) | ≥Moderate PVL: 6.6% vs. 13.4% (P=0.198)  Mean gradient (mmHg): 11.8±5.7 vs. 9.9±4.7 (P=0.037) | ≥Moderate PVL: 3.9% vs. 6.5% (P=0.688)  Mean gradient (mmHg): 10.8±5.2 vs. 10.3±4.7 (P=0.640) |
| **Single-center study**  Liao (2017) [2012-2017](55) | 87 vs. 70 | Type 0 56.3%, Type 1 43.7% | 42.5% vs. 35.7% (P=0.39) | 74.3 ± 6.4 vs. 74.3 ± 7.0 (P=0.39) | 7.9 ± 4.0% vs. TAV: 8.6 ± 4.4 (P = 0.27) | CoreValve and Venus-A | **All-cause death** |  |  | 9.2% vs. 4.3% (P=0.348) |  | Median follow-up 668 days:  12.6% vs 12.9% (P = 0.968) | |
|  |  |  |  |  |  |  | **Stroke** |  | 1.1% vs. 0 (P>0.99) |  |  |  | |
|  |  |  |  |  |  |  | **Hemodynamic & Echo outcomes** |  | ≥Moderate PVL: 1.2% vs. 0 (P>0.99)  Mean gradient (mmHg): 13 (9-16) vs. 12 (10-17) (P=0.934) |  |  |  | |
| *Outcomes in matching population are presented in PS-matching studies.  Ψ Period of publication of the studies included in the Meta-analysis  † Reported as KCCQ overall summary score.  ¥ In the non-matched population  AR = Aortic Regurgitation; ASD = absolute standardized difference; CI = confidence interval; HR = hazard ratio; NA= Not Applicable; PPM = Prosthesis-Patient-Mismatch; PS = propensity score; PVL = paravalvular leak; STS-PROM = Society of Thoracic Surgeons Predicted Risk of Mortality; TAVI = transcatheter aortic valve implantation; THV = transcathter heart valve (THV) | | | | | | | | | | | | | |

**Supplementary Table 5:** Transcatheter Aortic Valve Implantation **(**TAVI) in Bicuspid Aortic Valve (BAV) and Type of Transcatheter Heart Valve (THV)

| **Study**  (year of publication) [recruitment] | **N** | **Type of bicuspid valve** | **THVs being compared** | **Age (years)** mean±SD | **STS-PROM** (mean±SD or median (IQR)) | **Female** | **Outcomes** | **Follow-up** | | | | | | | | |
| --- | --- | --- | --- | --- | --- | --- | --- | --- | --- | --- | --- | --- | --- | --- | --- | --- |
| **Balloon-expandable (BE) THVs vs. Self-expanding (SE) THVs** |  |  |  |  |  |  |  | **(Peri)-procedural** | **In-hospital (discharge)** | **30-day** | **6-month** | **1-year** | **2-year** | **3-year** | **10-year** | |
| **Systemic Review & Meta-analysis**  Sá (2021)  8 observational studies§  [2013-2020] ψ(56) | 1’080 (620 BE THV; 460 SE THV) | Variable | BE THVs (Sapien/Sapien XT/Sapien 3)  vs.  SE THVs (CoreValve, Evolut R and Pro, Portico, and Venus A-Valve) | 77.2 | 4.6 | 41.0% | **All-cause death** | No significant difference (OR 0.86 [95% CI, 0.22–3.35], P = 0.749, I2 = 0%; very low quality of evidence) |  | No significant difference (OR 0.92 [95% CI, 0.54–1.57], P = 0.721, I2 = 0%; low quality of evi-dence) |  | No significant difference (OR 1.05 [95% CI, 0.48–2.29], P = 0.845, I2 = 0%; very low quality of evidence) |  |  |  | |
|  |  |  |  |  |  |  | **Stroke** | No significant difference (P = 0.178, I2 0%) |  |  |  |  |  |  |  | |
|  |  |  |  |  |  |  | **Hemodynamic & Echo outcomes** | ≥Moderate PVL:  OR 0.39 ([95% CI, 0.07–2.03], P = o.188, I2 = 77%, very low quality of evidence)  Subgroup analyses of ≥moderate PVL in 2nd generation BE THVs vs 2nd generation SE THVs: OR 0.08 ([95% CI, 0.02–0.35], P = 0.001, I2 = 0%, N = 2 studies) |  |  |  |  |  |  |  | |
| **Multi-center registry (29 centers in Europe, North America & Japan)**  Giacoppo (2025) [2007-2021](57) | 1’443 (860 BE THV; 583 SE THV) | Type 0 10.1%, Type 1 88.4%, Type 2 1.6% | BE THVs (Sapien XT, Sapien 3/3 Ultra) vs. SE THVs (Acurate neo, Allegra, Centera, Portico, CoreValve, Evolut R, Evolut Pro/Pro+) | 78.7 (72.6-83.2) | 3.2 (2.0–4.8) | 39.4% | **Death** |  | 1.2% vs. 2.2% (P=0.113, OR PSM 0.93 (0.45–1.93)) ¥ | 1.9% vs. 2.7% (P=0.350, OR PSM 0.82 (0.27–2.46)) ¥ |  | 7.6% vs. 7.4% (P=0.933, OR PSM 1.02 (0.51–2.04)) ¥ | 13.0 vs. 14.9% (P=0.473, OR PSM 1.09 (0.63–1.90)) ¥ | 20.8% vs. 22.0% (P=0.585, OR PSM 1.07 (0.63–1.80)) ¥ |  | |
|  |  |  |  |  |  |  | **Stroke** |  | 3.0 vs. 3.6% (P=0.549, OR PSM 1.09 (0.45–2.62)) ¥ | 3.4% vs. 3.8% (P=0.692, OR PSM 1.18 (0.51–2.77)) ¥ |  | 3.7% vs. 5.6% (P=0.205, OR PSM 0.88 (0.40–1.95)) ¥ | 3.7% vs. 8.1% (P=0.044, OR PSM 0.73 (0.35–1.51)) ¥ | 4.2% vs. 8.1% (P=0.056, OR PSM 0.75 (0.37–1.51)) ¥ |  | |
|  |  |  |  |  |  |  | **Aortic valve reintervention (**Repeat valve replacement) |  | Conversion to surgery: 0.2% vs. 0.7% (P=0.228, OR PSM 0.40, (0.04–4.19)) ¥  Additional THV implantation: 1.3% vs. 5.3% (P<0.001, OR PSM 0.37 (0.13–1.00)) ¥ | 0.4% vs. 1.3% (P=0.046, OR PSM 0.12 (0.00–4.24)) ¥ |  | 0.6% vs. 2.6% (P=0.003, OR PSM 0.37 (0.06–2.41)) ¥ | 1.1% vs. 3.1% (P=0.007, OR PSM 0.49 (0.02–9.75)) ¥ | 1.1% vs. 3.1% (P=0.007, OR PSM 0.49 (0.02–9.75)) ¥ |  | |
|  |  |  |  |  |  |  | **Valve-related Re-hospitalization** |  |  | 1.5% vs. 2.4% (P=0.272, OR PSM 0.65 (0.13–3.13)) ¥ |  | 4.2% vs. 9.6% (P<0.001, OR PSM 0.58 (0.17–1.90)) ¥ | 6.5% vs. 13.4% (P<0.001, OR PSM 0.63 (0.23–1.72)) ¥ | 7.9% vs. 16.1% (P<0.001, OR PSM 0.64 (0.25–1.69) ¥ |  | |
|  |  |  |  |  |  |  | **Annulus rupture** |  | 1.3% vs. 0.5% (P=0.145, OR PSM 6.85 (1.01–46.55)) ¥ |  |  |  |  |  |  | |
|  |  |  |  |  |  |  | **Permanent pacemaker implantation** |  | 11.0 vs. 17.8% (P<0.001, OR PSM 0.55 (0.33–0.90)) ¥ | 11.9% vs, 18.6% (P=0.001, OR PSM 0.56 (0.36–0.88)) ¥ | 13.9% vs. 20.7% (P=0.001, OR PSM 0.55 (0.36–0.85)) ¥ |  | 15.1% vs. 23.2% (P=0.001, OR PSM 0.55 (0.36–0.83)) ¥ | 15.5% vs. 25.1% (P<0.001, OR PSM 0.53 (0.35–0.81)) ¥ |  | |
|  |  |  |  |  |  |  | **Hemodynamic & Echo outcomes** |  | ≥Moderate AR: 3.3% vs. 12.7% (P<0.001, OR PSM 0.20 (0.08–0.48)) ¥  Mean gradient: 10.0 (8.0–13.0) vs. 8.0 (6.0–11.0) (P<0.001, OR PSM 1.69 (0.88–2.50)) ¥ | ≥Moderate AR: 3.3% vs. 12.7% (P<0.001, OR PSM 0.20 (0.08–0.48)) ¥  Mean gradient: 10.0 (8.0–13.0) vs. 8.0 (6.0–11.0) (P<0.001, OR PSM 1.69 (0.88–2.50)) ¥ |  |  |  |  |  | |
| **AD-HOC registry**  Buono (2024)  PS-matching population [2012-2019](58) | 602 (301 pairs) | Type 1 100.0% | Current (2^nd^ and 3^rd^) gen. BE THVs vs.  SE THVs | 78 (73-83) vs. 78 (72-82) (P = 0.760) | 2.55 (1.70-3.66) vs. 2.50 (1.59-3.86) (P = 0.790) | 36.9 % vs. 35.5% | **All-cause death** |  | 1.0% vs. 0.7% (P = 0.852) | 1.0% vs. 1.05 (P =0.928) |  | **Median 1.3**  **years [0.6-2.4 years]**  10.8% vs. 14.5% (P = 0.372) |  |  |  | |
|  |  |  |  |  |  |  | **Stroke** |  | 2.3% vs. 2.7% (P = 0.607) | 3.5% vs. 3.4% (P = 0.721) |  | 4.5% vs. 5.1% (P = 0.442) |  |  |  | |
|  |  |  |  |  |  |  | ***Re-hospitalization** |  |  | 0.4% vs 0.3% (no P value) |  | 2.4% vs. 2.7% (P = 0.844) |  |  | |  |
|  |  |  |  |  |  |  | **Aortic valve reintervention** | Procedural conversion to surgery: 1.3% vs 1.0% (P=0.991) |  | 0 vs. 0 |  | 1.0% vs. 0.7% (no P value) |  |  | |  |
|  |  |  |  |  |  |  | **Hemodynamic & Echo outcomes** |  | ≥Moderate PVL:  0.3% vs 7.0% (P =0.003)  Mean Gradient (mmHg):  11 [8-14] vs. 8 [6-11] (P<0.001) | ≥Moderate PVL:  1.7% vs 8.8% (P =0.001)  Mean Gradient (mmHg):  11 [8-14] vs. 8 [6-10] (P<0.001) |  |  |  |  | |  |
| **Five centers in Poland**  Gasecka (2022)  [2009-2017](45) | 130 (3 SE THVs; 96 SE THVs) | Not specified | BE THVs (old & new gen.) vs.  SE THVs (olde and new gen.) | 79 (74–82) | EuroScore II:  3.6 (2.6–5.1) | 40.0% | **All-cause death** |  |  |  |  |  |  |  | | 75.3% vs. 76.1% (P Log rank = 0.956; HR for SE THV 1.02, 95% CI:  0.52–1.99) |
| **BEAT registry**  Mangieri (2020)  PS-matching population [2013-2018](25) | 154 (77 pairs) | Type 0 9.7%, Type 1 62.3%, Type 2 0, Undetermined 27.3% | 3nd gen. BE Sapien 3 THV vs. SE Evolut R/PRO | 79.3 ± 7.8 | 4.3 ± 2.8 | 42.2% | **All-cause death** |  |  | 4.8% vs 3.0% (P = 0.452) |  | ~7% vs. ~10% vs (log-rank P-value = 0.507) |  |  | |  |
|  |  |  |  |  |  |  | **Stroke** |  |  | 0.9% vs 3.1% (P = 0.160) |  |  |  |  | |  |
|  |  |  |  |  |  |  | ***Re-hospitalization** |  |  | 2.3% vs 2.0% (P = 0.878) |  |  |  |  | |  |
|  |  |  |  |  |  |  | **Hemodynamic & Echo outcomes** |  |  | ≥ Moderate PVL:  0 vs. 9.3% (P = 0.043)  Mean gradient (mmHg): 11.5 vs. 8.5 (P<0.01) |  |  |  |  | |  |
| **Single-center study**  Boiago (2024**)** [before 2020]  (22) | 150 (67 BE THV vs. 83 SE THV) | Type 0 6.7%,  Type 1 92.6%, Type 2 0.7% | BE Sapien 3 vs. Various generation SE THVs of the CoreValve platform (CoreValve,Evolut R, Evolut PRO) | 80.5 ± 8.5 vs. 82.2 ± 6.4 (P=0.181) | 4.7 ± 3.1 vs. 6.0 ±8.1 | 48.0% | **All-cause death** | 1.5% vs. 1.2% (P = 1.000 |  | 3.1% vs. 3.7% (P = 1.000) |  | 14.0% vs. 12.0% (P = 0.730) |  | 28.0% vs. 28.1% (P = 0.988) | |  |
|  |  |  |  |  |  |  | **Stroke** |  |  | 1.5% vs. 2.4% (P = 1.000) |  | 5.3% vs. 6.7% (P = 1.000) |  | 6.0 vs 12.5% (p = 0.342 | |  |
|  |  |  |  |  |  |  | **Aortic valve reintervention** | Surgical conversion: 0 vs. 1.2% |  | 0 vs. 0 |  | 0 vs. 0 |  | 0 vs. 0 | |  |
|  |  |  |  |  |  |  | **Hemodynamic & Echo outcomes** | ≥Moderate PVL (angio): 3% vs. 7.2% |  | ≥ Moderate PVL:  9.5% vs. 10.1% (P =0.905)  Mean gradient (mmHg):  10.6 ± 5.4 vs. 8.5 ± 3.6 (P =0.010) |  | ≥ Moderate PVL:  5.4% vs. 17.2% (P =0.119)  Mean gradient (mmHg):  11.2 ± 4.8 vs. 8.6 ± 3.6 (P =0.004) |  | ≥ Moderate PVL (SEV 3/30 vs. BEV 0/25, P = 0.242)  Mean Gradient (mmHg):  10.7 ± 3.2 vs. 8.8 ± 3.8, (P = 0.063) | |  |
| **Multicenter study**  Jilaihawi (2016)§  [2005-2014](24) | 130 (70 BE THVs, 60 SE THVs) | **TAVI-directed classification**:  Tricommissural 23.5%, Bicommissural raphe-type 55.6%, Bicommissural non-raphe | BE Sapien  XT/3 vs. SE.  Corevalve | 76.2 ± 11.6 vs. 77.0 ±9 (P= 0.65) | 4.7 (2.8 -7.4) vs. 4.7 (3.3 -7.2) | 61.4% vs. 61.7% (P >0.99) | **All-cause death** | 1.4% vs. 1.7% (P>0.99) |  | 2.9% vs. 5.0% (P=0.66) | 4.9% vs. 7.9% (Lor-Rank P=0.63) |  |  |  | |  |
|  |  |  |  |  |  |  | **Stroke** |  |  | 4.5% vs. 1.7% (P=0.3) |  |  |  |  | |  |
|  |  |  |  |  |  |  | **Aortic valve reintervention** | Surgical conversion: 2.9% vs. 3.3% (P>0.99) |  |  |  |  |  |  | |  |
|  |  |  |  |  |  |  | **Hemodynamic & Echo outcomes** |  | Severe PVL 4.4% vs. 1.7% (P value for trend 0.27)  Mean gradient (mmHg):  10.0 (7.0-13.3) vs. 9.0 (7.0-13.0) (P=0.58) |  |  |  |  |  | |  |
| **Multicenter study**  Mylotte (2014)§  [2005-2014](30) | 139 (48 BE THVs , 91 SE THVs) | Type 0 26.7%, Type 1 68.3%, Type 2 5.0% | BE Sapien vs. SE Corevalve | 77.6 ± 9.7 vs. 78.2 ± 8.4 (P=0.71) | 5.0 ± 3.9 vs. 4.8 ± 3.1 (P=0.96) | 37.5% vs. 47.3% | **All-cause death** | 2.1% vs. 4.9% (P=0.66) |  | 6.3% vs 4.9% (P=0.69) | 14.6% vs. 6.6% (P=0.12) | 20.8% vs. 12.5% (P=0.12) |  |  | |  |
|  |  |  |  |  |  |  | **Stroke** |  |  | 2.1% vs. 2.2% (P=0.99) |  |  |  |  | |  |
| **Single-center study**  Deutsch (2023) [2015-2021](59) | 106  (68 BE THV; 38 SE THV) | Type 0 7.5%, Type 1 92.5%, Type 2 0%) | BE Sapien 3/3Ultra THV vs. SE Evolut R/PRO | 74.6 ± 8.8 vs.75.3 ± 8.7 (P = 0.670) | 2.6 ± 1.9 vs. 2.6 ± 1.6 (P = 0.374) | 35.0% | **All-cause death** |  |  | 0 vs. 2.6% (P = 0.358) |  |  |  |  | |  |
|  |  |  |  |  |  |  | **Stroke** |  |  | 2.9% vs. 2.6% (P = 0.547) |  |  |  |  | |  |
|  |  |  |  |  |  |  | **Hemodynamic & Echo outcomes** |  | Mild-moderate PVL at discharge:  7.4% vs. 13.2% (P = 0.305) (no patient with ≥moderate PVL) | Mean gradient (mmHg) at 30-day:  11.9 ± 4.6 vs. 9.2 ± 3.0 (P = 0.002) |  |  |  |  | |  |
| **Early generation THVs vs. Newer generation THVs** | **N** | **Type of bicuspid valve** | **THVs being compared** | **Age (years)** mean±SD | **STS-PROM** (mean±SD or median (IQR)) | **Female** | **Outcomes** | **(Peri)-procedural** | **In-hospital (discharge)** | **30-day** | **6-month** | **1-year** | **2-year** | **5-year** | | |
| **Bicuspid TAVR registry**  Yoon (2016)§  [2005-2015](29) | 301 (199 early generation THVs, 102 newer generation THVs) | Type 0 11.9%, Type 1 86.2%, Type 2 1.9%, undetermined 13.6% | Early gen. THVs (Sapien XT and CoreValve) vs. Newer gen. THVs (Sapien 3 and Lotus) | 77.0 ± 8.9 vs. 77.0 ± 9.8 (P =0.97) | 4.6 ± 5.1 vs. 4.9 ± 5.4 (P=0.57) | 42.5% | **All-cause death** | 1.5% vs 1.0% (P>0.99) |  | 4.5% vs. 3.9% (P>0.99) | 17.6% vs.  14.8% (log-rank P=0.49) |  |  |  | | |
|  |  |  |  |  |  |  | **Stroke** | 2.5% vs. 2.0% (P>0.99) |  |  |  |  |  |  | | |
|  |  |  |  |  |  |  | **Surgical conversion** | 4.0% vs 1.0% (P=0.27) |  |  |  |  |  |  | | |
|  |  |  |  |  |  |  | **Hemodynamic & Echo outcomes** | ≥ Moderate PVL: 8.5% vs. 0 (P=0.002) |  |  |  |  |  |  | | |
| **Five centers in Poland**  Gasecka (2022)  [2009-2017](45) | 130 (73 early generation THVs, 57 newer generation THVs) | Not specified | Early gen. THV (CoreValve, Boston Lotus, Edwards Sapien, Edwards Sapien XT) vs. Newer gen. (EvolutR, Symetis Accurate, Edwards Sapien 3) | 79 (72.5-83) vs. 79 (74.5-85) (P=0.395) | EuroScore II:  13.32% (7.22-24.07%) vs. 9.95% (5.62-16.7%) (P=0.109) | 39.7% vs. 40.3% (P=0.943) | **All-cause death** |  |  |  |  |  |  | 33.5.% vs. 25.4% (P Log rank = 0.0016, HR for newer generation : 0.27, 95% CI 0.12–0.62) | | |
| *Cardiac re-hospitalizations are reported and if not available, all-cause re-hospitalizations reported  § Included in the Meta-analysis by Sá et al. (2021)  Ψ Period of publication of the studies included in the Meta-analysis  ¥ Odd ratio (OR) in the Propensity-Score Matched (PSM) Population with 95% CI (Confidence Interval)  AR = Aortic Regurgitation; BE = Balloon-Expandable THV; NYHA = New-York Heart Association; OR = odds ratio; PSM=Propensity-Score Matching; PVL = paravalvular leak; SE = Self-Expanding THV; STS-PROM = Society of Thoracic Surgeons Predicted Risk of Mortality; TAVI = transcatheter aortic valve implantation. | | | | | | | | | | | | | | | | |

**Supplementary Table 6:** Transcatheter Aortic Valve Implantation **(**TAVI) and Bicuspid Aortic Valve (BAV) Phenotype

| **Study**  (year of publication) [recruitment] | **N** | **Type of bicuspid valve** | **Morphologies of bicuspid valve being compared** | **Female** | **Age (years)** mean±SD | **STS-PROM** (mean±SD or median (IQR)) | **Outcomes** | **Follow-up** | | | | |
| --- | --- | --- | --- | --- | --- | --- | --- | --- | --- | --- | --- | --- |
| **Comparison according to the calcification pattern** |  |  |  |  |  |  |  | **(Peri)-procedural** | **30-day** | **1-year** | **2-year** | **3-year** |
| **Multi-center study**  Yoon (2020) [2012-2019] (60) Ψ | 1’034 | Type 0 10.3%, Type 1 89.7% | No high-risk features vs. calcified raphe OR excessive leaflet calc. vs. calcified raphe AND excessive leaflet calc | 41.0% | 74.7±9.3 | 3.7±3.3 | **All-cause death** |  | 1.5% vs. 1.1% vs. 4.1% (P=0.016) | 3.8% vs. 4.6% vs. 13.6% (P<0.001) | 5.9% vs. 9.5% vs. 25.7% (P<0.001) |  |
|  |  |  |  |  |  |  | **Stroke** |  | 2.8% vs. 2.7% vs. 2.6% (P>0.99 |  |  |  |
|  |  |  |  |  |  |  | **Aortic valve reintervention** | Procedural conversion to surgery: 0.3% vs. 0.5% vs. 2.2% (P=0.028) |  |  | 0.9% vs. 0.6% vs. 0.4% (P=0.91) |  |
|  |  |  |  |  |  |  | **Hemodynamic & Echo outcomes** | ≥ Moderate PVL:  1.6% vs. 2.5% vs. 6.5% (P = 0.002) |  |  |  |  |
| **Single-center RESOLVE registry**  Nagasaka (2024)  [2016-2020](61)  (sub-group analysis) | 98 | Type 1 with excessive leaflet calc. 100.0% | No high-risk feature vs. calcified raphe OR opposite leaflet calc. vs.  calcified raphe AND opposite leaflet calc. | 25.0% vs. 25.8% vs. 20.0% (P=0.6) | 73.4±11.0 vs. 73.0±8.0 vs. 75.6±11.1 (P=0.941) | 3.7±2.8 vs. 3.2±2.3 vs. 3.5±3.6 (P=0.347) | **All-cause death** |  |  |  |  | Both high-risk features vs. No high-risk feature:  32.3% vs 7.9%, respectively (Log-rank P = 0.015)  One high-risk feature vs. No high-risk feature:  23.7% vs 7.9% (Log-rank  P = 0.073)  Overall (between the 3 groups): Log-rank P=0.051 |
| **Comparison according to THV underexpansion and eccentricity post-TAVI** | **N** | **Type of bicuspid valve** | **Morphologies of bicuspid valve being compared** | **Female** | **Age (years)** mean±SD | **STS-PROM** (mean±SD or median (IQR)) | **Outcomes** | **(Peri)-procedural** | **In-hospital (discharge)** | **30-day** | **2-year** | **3-year** |
| **Single-center RESOLVE registry**  Nagasaka (2024)  [2016-2020](61) | 229 | Type 0 17.0%, Type 1 83.0% | **Group 1** (n = 125), with no THV underexpansion or eccentricity; **Group 2** (n = 69), with underexpansion or eccentricity; and G**roup 3** (n = 35), with both | 40.0% vs. 39.1% vs. 37.1% (P=0.954) | 72.6 ±11.5 vs. 74.2 ± 8.5 vs. 73.1 ± 11.9 (P=0.773) | 3.01 ± 2.15 vs. 3.02 ± 2.18 vs. 3.20 ± 2.94 (P=0.71) | **All-cause death** |  |  | 0 vs. 0 vs. 2.9% (P=0.15) |  | 10.4% vs. 15.9% vs. 34.3% (P=0.003) |
|  |  |  |  |  |  |  | **Stroke** |  |  | 0.8% vs. 4.3% vs. 5.7% (P=0.10) |  | 4.8% vs. 4.3% vs. 11.4% (P=0.28) |
|  |  |  |  |  |  |  | **Heart Failure Re-Hospitalization** |  |  |  |  | 10.4% vs. 11.6% vs. 25.7% (P=0.039) |
|  |  |  |  |  |  |  | **Aortic valve reintervention** |  |  | 1.6% vs. 0 vs. 2.9 (P=0.27) |  |  |
|  |  |  |  |  |  |  | **Hemodynamic & Echo outcomes** |  |  | Mild to severe PVL: 20.0% vs. 24.6% vs. 31.4 (P=0.046)  No significant statistical difference in mean gradients (mmHg) | Mild to severe PVL: 32.0% vs. 37.7% vs  60.0% (P < 0.001)  No significant statistical difference in mean gradients (mmHg) |  |
|  |  |  |  |  |  |  | **CT outcomes** |  |  | HALT:  9.6% vs. 33.3% vs. 60.0% (P<0.001)  HAM, 4.8% vs. 24.6%  vs. 34.3% (P < 0.001) |  |  |
| **Comparison according to Jilaihawi’s classification** | **N** | **Type of bicuspid valve** | **Morphologies of bicuspid valve being compared** | **Female** | **Age (years)** mean±SD | **STS-PROM** (mean±SD or median (IQR)) | **Outcomes** | **(Peri)-procedural** | **In-hospital (discharge)** | **30-day** | **1-year** | **2-year** |
| **Multicenter study**  Jilaihawi (2016) [2005-2014](24) Ψ | 130 | **TAVI-directed classification**:  Tricommissural 23.5%, Bicommissural raphe-type 55.6%, Bicommissural non-raphe type 21.1% | Tricommissural vs. bicommissural | 38.5% | 76.6 ± 10.4 | 4.7 (3.0-7.3) | **All-cause death** | 0 vs. 2.0% (P>0.99) |  | 4.2% vs. 4.0% (P>0.99) |  |  |
|  |  |  |  |  |  |  | **Stroke** |  |  | 4.2% vs. 3.1% (P>0.99) |  |  |
|  |  |  |  |  |  |  | **Hemodynamic & Echo outcomes** |  | ≥ Moderate AR: 19% vs. 17.7% (P>0.99) |  |  |  |
|  |  |  | Bicommisural non-raphe type vs. Bicommissural raphe type | 38.5% | 76.6 ± 10.4 | 4.7 (3.0-7.3) | **All-cause death** | 9.5% vs. 0 (P=0.045) |  | 9.5% vs. 2.7% (P=0.21) |  |  |
|  |  |  |  |  |  |  | **Stroke** |  |  | 0 vs. 4.2% (P=0.39) |  |  |
|  |  |  |  |  |  |  | **Hemodynamic & Echo outcomes** |  | ≥ Moderate AR: 15.0% vs.19.4% (P>0.99) |  |  |  |
| **Comparison according to Sievers’ classification** | **N** | **Type of bicuspid valve** | **Morphologies of bicuspid valve being compared** | **Female** | **Age (years)** mean±SD | **STS-PROM** (mean±SD or median (IQR)) | **Outcomes** | **(Peri)-procedural** | **In-hospital (discharge)** | **30-day** | **1-year** | **5-year** |
| **Systematic review and meta-analysis** (9 retrospective non-matched studies)  Improta (2023) [2016-2022] Ψ (62) | 2’099 | Type 0 or 2 22.4%, Type 1 77.6%, Type 2 | Non-Type 1 vs. Type 1 |  |  |  | **All-cause death** |  |  | OR 1.38, 95% CI 0.48–4.03 (P = 0.55) |  |  |
|  |  |  |  |  |  |  | **Stroke** |  |  | OR 1.11, 95% CI 0.52–2.36 *(*P= 0.78) |  |  |
|  |  |  |  |  |  |  | **Permanent pacemaker implantation or new conduction anomlies** |  |  | OR 0.46, 95% CI 0.30–0.70 (P <0.001) |  |  |
|  |  |  |  |  |  |  | **Hemodynamic & Echo outcomes** |  |  | ≥ Moderate PVL: OR 0.75, 95% CI 0.42–1.34 (P = 0.33) |  |  |
| **Multi-cemter study**  Li (2025) [TAVI before 2018 and follow-up until 2023](63) | 134 BAV Type 0, 305 BAV Type 1, 2,114 TAV | Among BAV: Type 0 30.5%, Type 1 69.5% | Type 0 vs. Type 1 vs. TAV | 52.2% vs. 41.6% vs. 51.1% (P=0.007) | 72.9 ± 6.5 vs. 76.5 ± 7.4 vs. 80.8 ± 6.3 (P<0.001) | 3.4 [2.3-6.1] vs. 3.6 [2.2-5.6] vs. 3.6 [2.5-5.4] (P=0.08) | **All-cause death** |  |  | 2.5% vs. 4.3% vs. 4.5% |  | 11.0% vs. 34.5% vs. 45.5% (P<0.0001)  aHR Type 1 (Type 0 as a reference): 2.38 (95% CI: 1.32-4.28, P=0.004)  aHR TAV (Type 0 as a reference): 3.02 (95% CI 1.71-5.31, P<0.001) |
|  |  |  |  |  |  |  | **Hemodynamic & Echo outcomes** | ≥ Moderate PVL: 6.0% vs. 4.6% vs. 2.5% (P=0.011) |  |  |  |  |
| **Multicenter study**  Yousef (2015) [2005-2014](32) | 108 | Type 0 16.7%, Type 1 73.0%, Type 2 10.3% | Type 1 L-R vs. all other types of BAV | 32.1% | 75.5 ± 14.4 | 17.2 ± 12.2  (Logistic EuroScore) | **All-cause death** |  |  | 0 vs. 25.0% (P< 0.001) | 3.0% vs. 36.7% (P< 0.01) |  |
|  |  |  |  |  |  |  | **Stroke** |  |  |  |  |  |
|  |  |  |  |  |  |  | **Aortic valve reintervention** |  |  | 9.1% vs. 15.6% (P=0.61) |  |  |
|  |  |  |  |  |  |  | **Hemodynamic & Echo outcomes** |  |  | ≥ Moderate AR: 34.1% vs 41.4% (P = 0.70) | ≥ Moderate AR: 37.5% vs 26.7%  Mean gradient>20mmmHg: 8.3% vs. 5.9% |  |
| **Single-center study**  Lei (2019) [2012-2017](26) | 71 | Type 0 100% | Type 0 with mixed cusp fusion vs. Type 0 with coronary cusp fusion | 54.9% | 71.5 ± 6.1 vs. 72.6 ± 5.4 (P=0.44) | 6.9 ± 4.0 vs. 7.3 ± 3.0 (P=0.76) | **All-cause death** |  |  | 6.8% vs. 7.4% (P=1.000) | 6.8% vs. 11.1% (P=0.67) |  |
|  |  |  |  |  |  |  | **Stroke** |  | 4.5% vs. 3.7% (P=1.000) |  |  |  |
|  |  |  |  |  |  |  | **Hemodynamic & Echo outcomes** |  | ≥ Moderate PVL: 0 vs. 0  Mean gradient (mmHg):  15.9 ± 6.5 vs. 15.0 ± (P=0.57) |  |  |  |
| aHR = adjusted Hazard Ratio; AR = Aortic Regurgitation; HALT = Hypoattenuated Leaflet Thickening; HAM = Hypoattenuation Affecting Motion; NYHA = New-York Heart Association; PVL = Paravalvular Leak; STS-PROM = Society of Thoracic Surgeons Predicted Risk of Mortality; TAV = Tricuspid Aortic Valve; TAVI = transcatheter aortic valve implantation.  Ψ = Included in the Meta-Analysis from Improta et al.(62) | | | | | | | | | | | | |

**Supplementary Table 7:** Transcatheter Aortic Valve Implantation (TAVI) vs. Surgical Aortic Valve Replacement (SAVR) in Bicuspid Aortic Valve (BAV)

| **Study**  (year of publication) [recruitment] | **N TAVI vs. SAVR** | **Type of BAV** | **Female** | **Age** (mean±SD or median (IQR)) | **STS-PROM**  (mean±SD or median (IQR)) | **Type of THV device** | **Outcomes***  **(TAVI vs. SAVR)** | **Follow-up** | | | | | | |
| --- | --- | --- | --- | --- | --- | --- | --- | --- | --- | --- | --- | --- | --- | --- |
| **Randomized controlled trial (RCT)** |  |  |  |  |  |  |  | **(Peri)-procedural** | **In-hospital (discharge)** | **30-day** | **6-month** | **1-year** | **3-year** | **Long-term** |
| **Bicuspid subgroup of the NOTION-2 RCT**  Jorgensen (2024)  Jorgensen (2025)  [2016-2023](64, 65) | 49 vs. 51 | Type 0: 6.1% vs. 7.8%, Type 1: 91.8% vs. 92.2%, Type 2 2.1% vs. 0 | 44.9% vs. 37.1% | 69.7±3.6 vs. 70.0±3.4 | 1.0 (0.8-1.3) vs. 1.1 (0.8-1.5) | Evolut R/Pro/+ 55.1%, Sapien 3 26.5%, Portico/Navitor 6.1%, Acurate neo(2) 6.1%, Lotus 4.1%, Myval 2.0% | **All-cause death** |  |  |  |  | 4.1% vs. 2.0% (HR 2.1 [95% CI 0.2-22.8], P = 0.5) | π Absolute Risk difference: 2.2% (95% CI -6.4 to 10.8) |  |
|  |  |  |  |  |  |  | **Stroke** |  |  |  |  | 6.1% vs. 0  (P = 0.07) | π Absolute Risk difference: 6.2% (95% CI -2.4 to 14.8) |  |
|  |  |  |  |  |  |  | **Disabling stroke** |  |  |  |  | 2% vs. 0  (P =0.3) | π Absolute Risk difference: 2.0% (95% CI -1.9 to 6.0) |  |
|  |  |  |  |  |  |  | **Re-hospitalization** |  |  |  |  | 4.2% vs. 2.0% (HR 2.1 (95 % CI 0.2 to 23.5, P=0.5) | π Absolute Risk difference: 4.2% (95%CI -3.6 to 11.9) |  |
|  |  |  |  |  |  |  | **Aortic valve reintervention** |  |  |  |  | HR 2.1 [95% CI 0.2-23.5], P = 0.5 ¥ |  |  |
|  |  |  |  |  |  |  | **Functional outcomes** |  |  | †13 vs. 5 |  | †15 vs. 20 |  |  |
|  |  |  |  |  |  |  | **Hemodynamic & Echo outcomes** |  |  | Mean gradient (mmHg): 10.5 vs. 11.2 |  | Higher rate of  ≥Moderate PVL in TAVI vs. SAVR  (absolute risk difference of 9.1% (95% CI, 0.6%–17.6%))  Mean gradient (mmHg): 10.5 vs. 12.4 |  |  |
| **Systematic Review & Meta-analysis** | **N TAVI vs. SAVR** | **Type of BAV** | **Female** | **Age** (mean±SD or median (IQR)) | **STS-PROM**  (mean±SD or median (IQR)) | **Type of THV device** | **Outcomes***  **(TAVI vs. SAVR)** | **(Peri)-procedural** | **In-hospital (discharge)** | **30-day** | **6-month** | **1-year** | **2-year** | **Long-term** |
| **Systematic Review & Meta-analysis^§^** (6 retrospective studies)  Kang (2024**)** [2019-2022] Ψ (66) | 3’258 vs. 3’292 | Not specified | 38.7% vs. 37.3% | Not specified | Not specified | Not specified | **All-cause death** |  | OR 1.11 [95% CI 0.59–2.10], (P = 0.75) ¥ |  |  |  |  |  |
|  |  |  |  |  |  |  | **Stroke** |  | OR 1.2 [95%  CI 0.85–1.86] (P = 0.26) ¥ |  |  |  |  |  |
|  |  |  |  |  |  |  | **Hemodynamic & Echo outcomes** |  | PVL: OR 0.47; 95% CI 0.26–0.86 (P = 0.02) ¥ |  |  |  |  |  |
| **Propensity-Score matching studies** | **N TAVI vs. SAVR** | **Type of BAV** | **Female** | **Age** (mean±SD or median (IQR)) | **STS-PROM**  (mean±SD or median (IQR)) | **Type of THV device** | **Outcomes***  **(TAVI vs. SAVR)** |  |  |  |  |  |  |  |
| **Multi-centre** |  |  |  |  |  |  |  | **(Peri)-procedural** | **In-hospital (discharge)** | **30-day** | **6-month** | **1-year** | **2-year** | **3-year** |
| **Medicare and Medicaid database**  Chen (2023)  PS-matching study [2012-2019](67) | 797 vs. 797 | Not specified | 42.7% vs. 41.8% (SMD 1.8) | 73 (68-80) vs. 73 (69-77) (SMD 3.9) | Not specified | Not specified | **All-cause death** |  |  |  | 5.2% vs. 4.8%  HR 1.08, 95% CI, 0.67-  1.69 (P = 0.74) ¥ |  |  | 24.5% vs. 14.5%  HR for the risk between 6 months and 3 years post-intervention:  HR 2.16, 95% CI, 1.22-3.83 (P = 0.008) ¥ |
|  |  |  |  |  |  |  | **Stroke** |  |  | 2.4% vs. 2.3% (P = 0 .87) |  |  |  | HR 1.21, 95% CI, 0.75-1.96 ¥ |
|  |  |  |  |  |  |  | **Re-hospitalization** |  |  |  | HR, 0.51, 95% CI, 0.31-0.87 (P = 0.01) ¥ |  |  | 11.8% vs. 8.5% (P = 0.51)  HR for the risk between 6 months and 3 years post-intervention: HR, 4.78, 95% CI, 2.21-10.36 (P< 0.001). ¥ |
|  |  |  |  |  |  |  | **Aortic valve reintervention** |  |  |  |  |  |  | HR 1.03, 95% CI, 0.30-3.56 |
| **NRD study**  Majmundar (2022) **^§^**  PS-matching study [2016-2018](68) | 1’393 vs. 1’393 | Not specified | 37.8% vs. 38.4% (P=0.571) | 68.3 ±10.1 vs. 68.1 ±8.6 (P=0.790) | Not specified | Not specified | **All-cause death** |  | 0.7% vs 1.8%, (OR: 0.35 [95% CI 0.13-0.93], P = 0.035) |  |  |  |  |  |
|  |  |  |  |  |  |  | **Stroke** |  | 2.9% vs. 3.2% (P=0.717) |  | 3.0% vs. 3.9% (P = 0.206) |  |  |  |
|  |  |  |  |  |  |  | **Re-hospitalization** |  |  |  | 3.6% vs. 3.6% (HR 0.98 [95% CI 0.47-2.06], P = 0.966) |  |  |  |
|  |  |  |  |  |  |  | **Hemodynamic & Echo outcomes** |  | PVL: 0.9% vs. 0.6% (P=0.582) |  |  |  |  |  |
| **Medicare database**  Mentias (2020) **^§^**  PS-matching [2015-2017](69) | 699 vs. 699 | Not specified | Not specified | 74.7 ± 9.4 | Not specified | Not specified | **All-cause death** |  | 2.2% vs. 2.3% (P = 0.90) | 2.9% vs. 2.7% (P = 0.90) |  | 9.0% vs. 7.4%  (P = 0.34) | **Median follow-up of 631 days (IQ: 427 to 834 days):**  aHR: 1.08; 95% CI: 0.93 to 1.26 (P = 0.30) ¥ |  |
|  |  |  |  |  |  |  | **Stroke** |  | 2.7% vs. 2.9% (P = 0.90) |  |  | 4.0% vs. 3.7%  (P = 0.90) |  |  |
|  |  |  |  |  |  |  | **Re-hospitalization** |  |  |  |  | 2.3% vs. 2.9% (P = 0.60) |  |  |
| **NIS database**  Elbadawi (2019) **^§^**  PS-matching study [2012-2016](41) | 975 vs. 975 | Not specified | 40.0% vs 36.4% | 65.7 ± 16.5 vs. 65.2 ± 11.5 | Not specified | Not specified | **All-cause death** |  | 3.1% vs. 3.1%  (P >0.999) |  |  |  |  |  |
|  |  |  |  |  |  |  | **Stroke** |  | 2.1% vs. 2.6% (P=0.547) |  |  |  |  |  |
| **Finn TAVI Registry**  Husso (2021) **^§^**  PS-matching study [2008-2017](70) | 75 vs. 75 | Type 0 16.5%, Type 1 81.6%, Type 2 1.9% | 44.0% vs. 45.3% (P=0.870) | 75.8±8.4 vs. 75.7±6.3 | 2.9±1.7 vs. 3.1±3.2 (P=0.334) | CoreValve, CorValve Evolut, Lotus, Sapien XT, Sapien 3, Acurate neo | **All-cause death** |  |  | 1.3% vs. 5.3% (P=0.375) |  |  | 9.7% vs. 18.7%  (P = 0.268) |  |
|  |  |  |  |  |  |  | **Stroke** | 4.0% vs. 8.0% (P=0.508) |  |  |  |  |  |  |
|  |  |  |  |  |  |  | **Hemodynamic & Echo outcomes** | ≥Moderate PVL: 0 vs. 2.7% (P>0.99) |  |  |  |  |  |  |
| **NIS database**  Soud (2020) **^§^**  PS-matching study [2011-2014](71) | 68 vs. 68 | Not specified | 32.4% vs. 27.9% (P=0.709) | 65.0 ± 14.8 vs. 64.6 ± 12.4 (P=0.871) | Not specified | Not specified | **All-cause death** |  | 5.9% vs. 0 (P = 0.11) |  |  |  |  |  |
|  |  |  |  |  |  |  | **Stroke** |  | 1.5% vs. 0 (P = 1.00) |  |  |  |  |  |
|  |  |  |  |  |  |  | **Emergent cardiac surgery** |  | 14.7% vs. 17.6% (P=0.81) |  |  |  |  |  |
| **Inverse Probability Weighting (IPW) studies** | **N TAVI vs. SAVR** | **Type of BAV** | **Female** | **Age** (mean±SD or median (IQR)) | **STS-PROM**  (mean±SD or median (IQR)) | **Type of THV device** | **Outcomes***  **(TAVI vs. SAVR)** |  |  |  |  |  |  |  |
| **Multi-centre** |  |  |  |  |  |  |  | **(Peri)-procedural** | **In-hospital (discharge)** | **30-day** | **6-month** | **1-year** | **2-year** | **Long-term (≥4 years)** |
| **Medicare and Medicaid database**  Mehaffey (2024)  IPW analysis [2018-2022](72) | 3’166 vs. 8’123 | Not specified | 42.2% vs. 32.8% (P<0.001) | 71 (67-75) vs. 69 (66-72) (P<0.001) | 1.9 (0.9-3.1) vs. 1.7 (0.8-3.3) (P =0.038) | Not specified | **All-cause death** | OR 0.40 (P < 0.001) | HR 0.75 (0.579-0.98) (P=0.038) ¥ |  |  |  |  | **4-year**:  8.8% vs 5.7%  (HR 1.49 [95% CI 1.16-1.92], P <0.001) |
|  |  |  |  |  |  |  | **Stroke** |  | HR  1.78 (1.49-2.14) (P=0.001) ¥ | HR  1.29 (0.84-2.01) (P=0.246) ¥ |  |  |  | **4-year**:  2.4% vs 1.5% (HR 1.35 [95% CI 1.21-1.50], P < 0.001) |
|  |  |  |  |  |  |  | **Re-hospitalization** |  |  | HR 0.47 (0.41-0.55) (P=0.001) |  |  |  | **5-year** (accounting for the 30-day blanking period): HR, 1.23 [95% CI 1.01-1.51]; P = 0.045) ¥ |
|  |  |  |  |  |  |  | **Aortic valve reintervention** |  |  |  |  |  |  | HR 0.64 (0.396-1.03) (P=0 .072) ¥ |
| **NRD study**  Sanaiha (2023)  IPW analysis [2012-2019](73) | 3’855 vs. 52’476 | Not specified | 38.8% vs. 30.8% (P<0.001) | 69 (62-76) vs. 59 (51-66) (P<0.001) | Not specified | Not specified | **All-cause death** |  | 1.9% vs. 1.6%  (P = 0.49) |  |  |  |  |  |
|  |  |  |  |  |  |  | **Stroke** |  | 1.1% vs. 0.9%  (P = 0.26) |  |  |  |  |  |
| **Non-adjusted study** | **N TAVI vs. SAVR** | **Type of BAV** | **Female** | **Age** (mean±SD or median (IQR)) | **STS-PROM**  (mean±SD or median (IQR)) | **Type of THV device** | **Outcomes***  **(TAVI vs. SAVR)** | **(Peri)-procedural** | **In-hospital (discharge)** | **30-day** | **6-month** | **1-year** | **2-year** | **2.86 ± 1.47 years** |
| **Single-center study**  Tsai (2021) **^§^**  [2013-2018](74) | 48 vs. 82 | NA | 41.7% vs. 31.5% | 65.7 ± 7.8 vs. 51.5 ± 32.3 | 6.3 ± 6.4 vs. 1.6 ± 1.7 (P<0.05) | Not specified | **All-cause death** |  |  |  |  |  |  | 12.5% vs. 4.9% |
|  |  |  |  |  |  |  | **Stroke** |  |  |  |  |  |  | 2.1% vs. 0 |
|  |  |  |  |  |  |  | **Hemodynamic & Echo outcomes** |  |  |  |  |  |  | ≥Moderate PVL: 12.5% vs. 0 |
| π Absolute Risk Difference = Event Rate in the TAVI group – Event Rate in the SAVR group  *****Outcomes in matching population are presented in PS-matching or IPW study.  † Reported as mean KCCQ score change relative to baseline  § Included in the Meta-analysis by Kang et al. (2024)  Ψ Period of publication of the studies included in the Meta-analysis  ¥ OR, HR and aHR are shown for TAVI compared with SAVR  aHR = adjusted hazard ratio; HFH = Heart Failure (Re-)Hospitalization; HR = hazard ratio; NIS = National Inpatient Sample; NRD = Nationwide Readmission Database; IPW = inverse probability weighting; OR = odds ratio; PS = propensity score; PVL = paravalvular leak; SAVR = surgical aortic valve replacement; SMD = standardized mean difference; STS-PROM = Society of Thoracic Surgeons Predicted Risk of Mortality; TAVI = transcatheter aortic valve implantation. | | | | | | | | | | | | | | |

**References**

1. Huntley GD, Thaden JJ, Alsidawi S, Michelena HI, Maleszewski JJ, Edwards WD, et al. Comparative study of bicuspid vs. tricuspid aortic valve stenosis. European heart journal Cardiovascular Imaging. 2018;19(1):3-8.

2. Holmgren A, Enger TB, Naslund U, Videm V, Valle S, Evjemo KJD, et al. Long-term results after aortic valve replacement for bicuspid or tricuspid valve morphology in a Swedish population. Eur J Cardiothorac Surg. 2021;59(3):570-6.

3. Haunschild J, Misfeld M, Schroeter T, Lindemann F, Davierwala P, von Aspern K, et al. Prevalence of permanent pacemaker implantation after conventional aortic valve replacement-a propensity-matched analysis in patients with a bicuspid or tricuspid aortic valve: a benchmark for transcatheter aortic valve replacement. Eur J Cardiothorac Surg. 2020;58(1):130-7.

4. Coti I, Werner P, Kaider A, Mach M, Kocher A, Laufer G, et al. Rapid-deployment aortic valve replacement for patients with bicuspid aortic valve: a single-centre experience. Eur J Cardiothorac Surg. 2022;62(4).

5. Celik M, Milojevic M, Durko AP, Oei FBS, Bogers A, Mahtab EAF. Differences in baseline characteristics and outcomes of bicuspid and tricuspid aortic valves in surgical aortic valve replacement. Eur J Cardiothorac Surg. 2021;59(6):1191-9.

6. Wedin JO, Vedin O, Rodin S, Simonson OE, Horsne Malmborg J, Pallin J, et al. Patients With Bicuspid Aortic Stenosis Demonstrate Adverse Left Ventricular Remodeling and Impaired Cardiac Function Before Surgery With Increased Risk of Postoperative Heart Failure. Circulation. 2022;146(17):1310-22.

7. Hirji SA, Wegermann Z, Vemulapalli S, Newell P, Grau-Sepulveda M, O'Brien S, et al. Benchmarking Outcomes of Surgical Aortic Valve Replacement in Patients With Bicuspid Aortic Valves. Ann Thorac Surg. 2023;116(6):1222-31.

8. Im S, Kim KH, Sohn SH, Kang Y, Kim JS, Choi JW. Comparable Outcomes of Bicuspid Aortic Valves for Rapid-Deployment Aortic Valve Replacement. J Chest Surg. 2023;56(6):435-44.

9. Makkinejad A, Satija D, Monaghan K, Kim K, Fukuhara S, Patel HJ, et al. The Impact of Bicuspid Aortic Valve on Long-term Outcomes After Bioprosthetic Valve Replacement. Ann Thorac Surg. 2025;119(4):852-60.

10. Rinewalt D, McCarthy PM, Malaisrie SC, Fedak PW, Andrei AC, Puthumana JJ, et al. Effect of aortic aneurysm replacement on outcomes after bicuspid aortic valve surgery: validation of contemporary guidelines. J Thorac Cardiovasc Surg. 2014;148(5):2060-9.

11. Svensson LG, Kim KH, Blackstone EH, Rajeswaran J, Gillinov AM, Mihaljevic T, et al. Bicuspid aortic valve surgery with proactive ascending aorta repair. J Thorac Cardiovasc Surg. 2011;142(3):622-9, 9 e1-3.

12. Kaneko T, Shekar P, Ivkovic V, Longford NT, Huang CC, Sigurdsson MI, et al. Should the dilated ascending aorta be repaired at the time of bicuspid aortic valve replacement? Eur J Cardiothorac Surg. 2018;53(3):560-8.

13. Celik M, Mahtab EAF, Bogers A. Surgical Aortic Valve Replacement with Concomitant Aortic Surgery in Patients with Purely Bicuspid Aortic Valve and Associated Aortopathy. J Cardiovasc Dev Dis. 2021;8(2).

14. Brown B, Le T, Naeem A, Malik A, Norton EL, Wu X, et al. Stentless valves for bicuspid and tricuspid aortic valve disease. JTCVS Open. 2021;8:177-88.

15. Jia Y, Maznyczka A, Boiago M, Khokhar A, Tomii D, Neylon A, et al. Long-Term Durability of Transcatheter Aortic Valves in Patients With Bicuspid Aortic Stenosis. Catheter Cardiovasc Interv. 2025.

16. Zahr F, Ramlawi B, Reardon MJ, Deeb GM, Yakubov SJ, Song HK, et al. 3-Year Outcomes From the Evolut Low Risk TAVR Bicuspid Study. JACC Cardiovascular interventions. 2024;17(14):1667-75.

17. Forrest JK, Ramlawi B, Deeb GM, Zahr F, Song HK, Kleiman NS, et al. Transcatheter Aortic Valve Replacement in Low-risk Patients With Bicuspid Aortic Valve Stenosis. JAMA Cardiol. 2021;6(1):50-7.

18. Tchétché D, Ziviello F, De Biase C, De Backer O, Hovasse T, Leroux L, et al. Transcatheter aortic valve implantation with the Evolut platform for bicuspid aortic valve stenosis: the international, multicentre, prospective BIVOLUTX registry. EuroIntervention. 2023;19(6):502-11.

19. Williams MR, Jilaihawi H, Makkar R, O'Neill WW, Guyton R, Malaisrie SC, et al. The PARTNER 3 Bicuspid Registry for Transcatheter Aortic Valve Replacement in Low-Surgical-Risk Patients. JACC Cardiovascular interventions. 2022;15(5):523-32.

20. Waksman R, Craig PE, Torguson R, Asch FM, Weissman G, Ruiz D, et al. Transcatheter Aortic Valve Replacement in Low-Risk Patients With Symptomatic Severe Bicuspid Aortic Valve Stenosis. JACC Cardiovascular interventions. 2020;13(9):1019-27.

21. Attinger-Toller A, Bhindi R, Perlman GY, Murdoch D, Weir-McCall J, Blanke P, et al. Mid-term outcome in patients with bicuspid aortic valve stenosis following transcatheter aortic valve replacement with a current generation device: A multicenter study. Catheter Cardiovasc Interv. 2020;95(6):1186-92.

22. Boiago M, Bellamoli M, De Biase C, Beneduce A, Alonso LG, Laforgia P, et al. Three-year clinical outcomes after transcatheter aortic valve implantation in patients with bicuspid aortic disease: Comparison between self-expanding and balloon-expandable valves. Catheter Cardiovasc Interv. 2024;103(6):1004-14.

23. Fiorina C, Massussi M, Ancona M, Montorfano M, Petronio AS, Tarantini G, et al. Mid-term outcomes and hemodynamic performance of transcatheter aortic valve implantation in bicuspid aortic valve stenosis: Insights from the bicuSpid TAvi duraBILITY (STABILITY) registry. Catheter Cardiovasc Interv. 2023;102(6):1132-9.

24. Jilaihawi H, Chen M, Webb J, Himbert D, Ruiz CE, Rodés-Cabau J, et al. A Bicuspid Aortic Valve Imaging Classification for the TAVR Era. JACC Cardiovascular imaging. 2016;9(10):1145-58.

25. Mangieri A, Tchetchè D, Kim WK, Pagnesi M, Sinning JM, Landes U, et al. Balloon Versus Self-Expandable Valve for the Treatment of Bicuspid Aortic Valve Stenosis: Insights From the BEAT International Collaborative Registrys. Circ Cardiovasc Interv. 2020;13(7):e008714.

26. Lei WH, Liao YB, Wang ZJ, Ou YW, Tsauo JY, Li YJ, et al. Transcatheter Aortic Valve Replacement in Patients with Aortic Stenosis Having Coronary Cusp Fusion versus Mixed Cusp Fusion Nonraphe Bicuspid Aortic Valve. J Interv Cardiol. 2019;2019:7348964.

27. Yoon SH, Sharma R, Chakravarty T, Kawamori H, Maeno Y, Miyasaka M, et al. Clinical outcomes and prognostic factors of transcatheter aortic valve implantation in bicuspid aortic valve patients. Ann Cardiothorac Surg. 2017;6(5):463-72.

28. Perlman GY, Blanke P, Dvir D, Pache G, Modine T, Barbanti M, et al. Bicuspid Aortic Valve Stenosis: Favorable Early Outcomes With a Next-Generation Transcatheter Heart Valve in a Multicenter Study. JACC Cardiovascular interventions. 2016;9(8):817-24.

29. Yoon SH, Lefèvre T, Ahn JM, Perlman GY, Dvir D, Latib A, et al. Transcatheter Aortic Valve Replacement With Early- and New-Generation Devices in Bicuspid Aortic Valve Stenosis. Journal of the American College of Cardiology. 2016;68(11):1195-205.

30. Mylotte D, Lefevre T, Sondergaard L, Watanabe Y, Modine T, Dvir D, et al. Transcatheter aortic valve replacement in bicuspid aortic valve disease. Journal of the American College of Cardiology. 2014;64(22):2330-9.

31. Kochman J, Zbroński K, Kołtowski Ł, Parma R, Ochała A, Huczek Z, et al. Transcatheter aortic valve implantation in patients with bicuspid aortic valve stenosis utilizing the next-generation fully retrievable and repositionable valve system: mid-term results from a prospective multicentre registry. Clin Res Cardiol. 2020;109(5):570-80.

32. Yousef A, Simard T, Webb J, Rodés-Cabau J, Costopoulos C, Kochman J, et al. Transcatheter aortic valve implantation in patients with bicuspid aortic valve: A patient level multi-center analysis. International journal of cardiology. 2015;189:282-8.

33. Saeed Al-Asad K, Martinez Salazar A, Radwan Y, Wang E, Salam MF, Sabanci R, et al. Transcatheter Aortic Valve Replacement in Bicuspid Versus Tricuspid Aortic Valve Stenosis: Meta-Analysis and Systemic Review. The American journal of cardiology. 2023;203:105-12.

34. Montalto C, Sticchi A, Crimi G, Laricchia A, Khokhar AA, Giannini F, et al. Outcomes After Transcatheter Aortic Valve Replacement in Bicuspid Versus Tricuspid Anatomy: A Systematic Review and Meta-Analysis. JACC Cardiovascular interventions. 2021;14(19):2144-55.

35. Sá MP, Van den Eynde J, Jacquemyn X, Tasoudis P, Erten O, Dokollari A, et al. Late outcomes of transcatheter aortic valve implantation in bicuspid versus tricuspid valves: Meta-analysis of reconstructed time-to-event data. Trends Cardiovasc Med. 2023;33(7):458-67.

36. Yamanaka F, Shishido K, Moriyama N, Ochiai T, Miyashita H, Yokoyama H, et al. Incidence and Prognosis of Prosthesis-Patient Mismatch After Transcatheter Aortic Valve Replacement for Bicuspid Aortic Stenosis. JACC Cardiovascular interventions. 2025;18(4):492-502.

37. Deeb GM, Reardon MJ, Ramlawi B, Yakubov SJ, Chetcuti SJ, Kleiman NS, et al. Propensity-Matched 1-Year Outcomes Following Transcatheter Aortic Valve Replacement in Low-Risk Bicuspid and Tricuspid Patients. JACC Cardiovascular interventions. 2022;15(5):511-22.

38. Makkar RR, Yoon SH, Chakravarty T, Kapadia SR, Krishnaswamy A, Shah PB, et al. Association Between Transcatheter Aortic Valve Replacement for Bicuspid vs Tricuspid Aortic Stenosis and Mortality or Stroke Among Patients at Low Surgical Risk. JAMA. 2021;326(11):1034-44.

39. Forrest JK, Kaple RK, Ramlawi B, Gleason TG, Meduri CU, Yakubov SJ, et al. Transcatheter Aortic Valve Replacement in Bicuspid Versus Tricuspid Aortic Valves From the STS/ACC TVT Registry. JACC Cardiovascular interventions. 2020;13(15):1749-59.

40. Makkar RR, Yoon SH, Leon MB, Chakravarty T, Rinaldi M, Shah PB, et al. Association Between Transcatheter Aortic Valve Replacement for Bicuspid vs Tricuspid Aortic Stenosis and Mortality or Stroke. Jama. 2019;321(22):2193-202.

41. Elbadawi A, Saad M, Elgendy IY, Barssoum K, Omer MA, Soliman A, et al. Temporal Trends and Outcomes of Transcatheter Versus Surgical Aortic Valve Replacement for Bicuspid Aortic Valve Stenosis. JACC Cardiovascular interventions. 2019;12(18):1811-22.

42. Nagaraja V, Suh W, Fischman DL, Banning A, Martinez SC, Potts J, et al. Transcatheter aortic valve replacement outcomes in bicuspid compared to trileaflet aortic valves. Cardiovasc Revasc Med. 2019;20(1):50-6.

43. Mangieri A, Chieffo A, Kim WK, Stefanini GG, Rescigno G, Barbanti M, et al. Transcatheter aortic valve implantation using the ACURATE neo in bicuspid and tricuspid aortic valve stenosis: a propensity-matched analysis of a European experience. EuroIntervention. 2018;14(12):e1269-e75.

44. Yoon SH, Bleiziffer S, De Backer O, Delgado V, Arai T, Ziegelmueller J, et al. Outcomes in Transcatheter Aortic Valve Replacement for Bicuspid Versus Tricuspid Aortic Valve Stenosis. Journal of the American College of Cardiology. 2017;69(21):2579-89.

45. Gasecka A, Walczewski M, Witkowski A, Dabrowski M, Huczek Z, Wilimski R, et al. Long-Term Mortality After TAVI for Bicuspid vs. Tricuspid Aortic Stenosis: A Propensity-Matched Multicentre Cohort Study. Frontiers in cardiovascular medicine. 2022;9:894497.

46. He J, Xiong TY, Yao YJ, Peng Y, Wei JF, Zhao ZG, et al. Outcomes Following Transcatheter Aortic Valve Replacement for Aortic Stenosis in Patients With Type 0 Bicuspid, Type 1 Bicuspid, and Tricuspid Aortic Valves. Circ Cardiovasc Interv. 2023;16(12):e013083.

47. Michel JM, Frangieh AH, Giacoppo D, Alvarez-Covarrubias HA, Pellegrini C, Rheude T, et al. Safety and efficacy of minimalist transcatheter aortic valve implantation using a new-generation balloon-expandable transcatheter heart valve in bicuspid and tricuspid aortic valves. Clin Res Cardiol. 2021;110(12):1993-2006.

48. De Biase C, Mastrokostopoulos A, Philippart R, Desroche LM, Blanco S, Rehal K, et al. Aortic valve anatomy and outcomes after transcatheter aortic valve implantation in bicuspid aortic valves. International journal of cardiology. 2018;266:56-60.

49. Pineda AM, Rymer J, Wang A, Banks AZ, Koweek LH, Plichta R, et al. Transcatheter aortic valve replacement for patients with severe bicuspid aortic stenosis. Am Heart J. 2020;224:105-12.

50. Halim SA, Edwards FH, Dai D, Li Z, Mack MJ, Holmes DR, et al. Outcomes of Transcatheter Aortic Valve Replacement in Patients With Bicuspid Aortic Valve Disease: A Report From the Society of Thoracic Surgeons/American College of Cardiology Transcatheter Valve Therapy Registry. Circulation. 2020;141(13):1071-9.

51. Tchetche D, de Biase C, van Gils L, Parma R, Ochala A, Lefevre T, et al. Bicuspid Aortic Valve Anatomy and Relationship With Devices: The BAVARD Multicenter Registry. Circ Cardiovasc Interv. 2019;12(1):e007107.

52. Sannino A, Cedars A, Stoler RC, Szerlip M, Mack MJ, Grayburn PA. Comparison of Efficacy and Safety of Transcatheter Aortic Valve Implantation in Patients With Bicuspid Versus Tricuspid Aortic Valves. The American journal of cardiology. 2017;120(9):1601-6.

53. Jin Q, Chen S, Yang X, Li M, Li W, Zhang X, et al. Clinical outcomes of bicuspid versus tricuspid aortic valve stenosis after transcatheter aortic valve replacement with self-expandable valves. BMC Cardiovasc Disord. 2022;22(1):540.

54. Zhou D, Yidilisi A, Fan J, Zhang Y, Dai H, Zhu G, et al. Three-year outcomes of transcatheter aortic valve implantation for bicuspid versus tricuspid aortic stenosis. EuroIntervention. 2022;18(3):193-202.

55. Liao YB, Li YJ, Xiong TY, Ou YW, Lv WY, He JL, et al. Comparison of procedural, clinical and valve performance results of transcatheter aortic valve replacement in patients with bicuspid versus tricuspid aortic stenosis. International journal of cardiology. 2018;254:69-74.

56. Sá M, Simonato M, Van den Eynde J, Cavalcanti LRP, Alsagheir A, Tzani A, et al. Balloon versus self-expandable transcatheter aortic valve implantation for bicuspid aortic valve stenosis: A meta-analysis of observational studies. Catheter Cardiovasc Interv. 2021;98(5):E746-e57.

57. Giacoppo D, Alvarez-Covarrubias H, Xhepa E, Matsuda Y, Cangemi S, Michel JM, et al. Transcatheter Aortic Valve Replacement With Balloon- Versus Self-Expandable Bioprostheses for the Treatment of Bicuspid Aortic Valve Stenosis. Circulation. 2025.

58. Buono A, Zito A, Kim WK, Fabris T, De Biase C, Bellamoli M, et al. Balloon-Expandable vs Self-Expanding Valves for Transcatheter Treatment of Sievers Type 1 Bicuspid Aortic Stenosis. JACC Cardiovascular interventions. 2024.

59. Deutsch O, Vitanova K, Ruge H, Erlebach M, Krane M, Lange R. Results of new-generation balloon vs. self-expandable transcatheter heart valves for bicuspid aortic valve stenosis. Frontiers in cardiovascular medicine. 2023;10:1252163.

60. Yoon SH, Kim WK, Dhoble A, Milhorini Pio S, Babaliaros V, Jilaihawi H, et al. Bicuspid Aortic Valve Morphology and Outcomes After Transcatheter Aortic Valve Replacement. Journal of the American College of Cardiology. 2020;76(9):1018-30.

61. Nagasaka T, Patel V, Shechter A, Suruga K, Koren O, Chakravarty T, et al. Impact of Balloon-Expandable TAVR Valve Deformation and Calcium Distribution on Outcomes in Bicuspid Aortic Valve. JACC Cardiovascular interventions. 2024;17(17):2023-37.

62. Improta R, Di Pietro G, Kola N, Birtolo LI, Colantonio R, Bruno E, et al. A Meta-Analysis of Short-Term Outcomes of TAVR versus SAVR in Bicuspid Aortic Valve Stenosis and TAVR Results in Different Bicuspid Valve Anatomies. J Clin Med. 2023;12(23).

63. Li W, Jia Y, Li H, Kobari Y, Li J, Feng Y, et al. Long-Term Outcomes of BAV-0 Patients Compared With BAV-1 and TAV Patients After TAVR. JACC Cardiovascular interventions. 2025.

64. Jørgensen TH, Thyregod HGH, Savontaus M, Willemen Y, Bleie Ø, Tang M, et al. Transcatheter Aortic Valve Implantation in Low-Risk Tricuspid or Bicuspid Aortic Stenosis: The NOTION-2 Trial. European heart journal. 2024.

65. Jørgensen TH, Savontaus M, Willemen Y, Bleie Ø, Tang M, Angerås O, et al. Three-Year-Follow-Up of the NOTION-2 Trial: TAVR Versus SAVR to Treat Younger Low-Risk Patients with Tricuspid or Bicuspid Aortic Stenosis. Circulation. 2025.

66. Kang JJ, Fialka NM, El-Andari R, Watkins A, Hong Y, Mathew A, et al. Surgical vs transcatheter aortic valve replacement in bicuspid aortic valve stenosis: A systematic review and meta-analysis. Trends Cardiovasc Med. 2023.

67. Chen Q, Malas J, Megna D, Tam DY, Gill G, Rowe G, et al. Bicuspid aortic stenosis: National three-year outcomes of transcatheter versus surgical aortic valve replacement among Medicare beneficiaries. The Journal of thoracic and cardiovascular surgery. 2023.

68. Majmundar M, Kumar A, Doshi R, Shariff M, Krishnaswamy A, Reed GW, et al. Early outcomes of transcatheter versus surgical aortic valve implantation in patients with bicuspid aortic valve stenosis. EuroIntervention. 2022;18(1):23-32.

69. Mentias A, Sarrazin MV, Desai MY, Saad M, Horwitz PA, Kapadia S, et al. Transcatheter Versus Surgical Aortic Valve Replacement in Patients With Bicuspid Aortic Valve Stenosis. Journal of the American College of Cardiology. 2020;75(19):2518-9.

70. Husso A, Airaksinen J, Juvonen T, Laine M, Dahlbacka S, Virtanen M, et al. Transcatheter and surgical aortic valve replacement in patients with bicuspid aortic valve. Clin Res Cardiol. 2021;110(3):429-39.

71. Soud M, Al-Khadra Y, Darmoch F, Moussa Pacha H, Fanari Z, Alraies MC. Transcatheter aortic valve replacement in patients with bicuspid aortic valve stenosis: national trends and in-hospital outcomes. Avicenna J Med. 2020;10(1):22-8.

72. Mehaffey JH, Jagadeesan V, Kawsara M, Hayanga JWA, Chauhan D, Wei L, et al. Transcatheter vs Surgical Aortic Valve Replacement in Bicuspid Aortic Valves. Ann Thorac Surg. 2024.

73. Sanaiha Y, Hadaya JE, Tran Z, Shemin RJ, Benharash P. Transcatheter and Surgical Aortic Valve Replacement in Patients With Bicuspid Aortic Valve Stenosis. Ann Thorac Surg. 2023;115(3):611-8.

74. Tsai HY, Lin YS, Wu IC, Kuo LY, Chen BY, Shen SL, et al. Major adverse cardiac events and functional capacity in patients at intermediate risk undergoing transcatheter versus surgical aortic valve replacement for aortic stenosis with bicuspid valves. J Card Surg. 2021;36(3):828-33.
